# Invariant visual object and face learning in the ventral cortical visual pathway: A biologically plausible model

**DOI:** 10.1371/journal.pcbi.1013959

**Published:** 2026-02-11

**Authors:** Chenfei Zhang, Edmund T. Rolls, Jianfeng Feng

**Affiliations:** 1 Institute for the Science and Technology of Brain-Inspired Intelligence, Fudan University, Shanghai, China; 2 Oxford Centre for Computational Neuroscience, Oxford, United Kingdom; 3 University of Warwick, Department of Computer Science, Coventry, United Kingdom; Soochow University, CHINA

## Abstract

How transform-invariant visual representations of objects and faces are learned in the ventral visual cortical pathway is a massive computational problem. Here we describe key advances towards a biologically plausible four-layer network that performs these computations from the primary visual cortex to the inferior temporal visual cortex. The architecture is a four-layer competitive network with layer-to-layer convergence using a short-term memory trace local synaptic learning rule to associate transforming inputs from an object during natural viewing. The key advances towards biological plausibility include: (1) a synaptic modification rule including long-term depression dependent on synaptic strength instead of artificial synaptic weight normalization; (2) limiting the strength of synapses promotes distributed weights, improving transform-invariant learning; (3) reducing the ability of low firing rate neurons to participate in learning analogous to the NMDA receptor non-linearity can increase the storage capacity; (4) demonstrated network scalability towards high capacity. These advances have many implications for better understanding of cortical computations. These advances in biological plausibility of this approach are compared with artificial networks of the same ventral cortical processing stream that do not use a local synaptic learning rule and are less biologically plausible, and implications for AI models are described.

## Author summary

The ventral visual cortical stream of primates including human computes how to represent faces and objects and to identify them from any view. Here we describe a biologically plausible model of this cortical processing in a four-layer model from the primary visual cortex to the inferior temporal visual cortex. The model forms combinations of features from stage to stage of the hierarchy, and learns view invariant representations by using a slow synaptic learning rule that helps to associate together different views of an object while it transforms from view to view. Here we introduce a biologically plausible learning rule that increases synaptic strengths if there is postsynaptic and pre-synaptic activity, but decreases the synaptic strength if there is postsynaptic but little presynaptic activity, with the decrease being greater if the synaptic weight is strong. This, and clipping the synaptic weights to a biologically plausible maximum value, help to improve performance so that hundreds of objects or faces can be trained in a network of modest size (256 x 256 neurons in each layer, with 1000 synapses on each neuron). This provides a biologically plausible model of how humans and other primates learn view-invariant representations of objects and faces, and is contrasted with AI approaches.

## 1. Introduction

Rolls and colleagues discovered neurons in the inferior temporal visual cortex with transform-invariant responses to faces and objects [1–7]. The neurons have representations that are remarkably invariant with respect to translation, size, contrast, spatial frequency, viewing distance, and in some cases view [3,8–13]. Transform invariance is important as a neuronal output of the object and face cortical visual system, for then structures that learn associations of these faces and objects with other stimuli such as reward for the orbitofrontal cortex and amygdala, and viewed location for the hippocampus, will generalize correctly to the same face or object if it is seen later in a different view or other transform. These discoveries [5,7,9,14], and discoveries on how these neurons code for faces and objects [15], have been followed up in many subsequent investigations [16–34]. In more recent work, we have followed these pathways using MRI and MEG connectivity analyses in the human ventrolateral cortical visual stream to posterior inferior temporal visual cortex regions FFC, TE2p and TE1p [35–37], and have shown that not only are these regions activated by faces and objects such as tools and body parts, but that further cortical regions are activated that represent the semantic properties of the objects, such as that they move [38].

**Fig 1:**
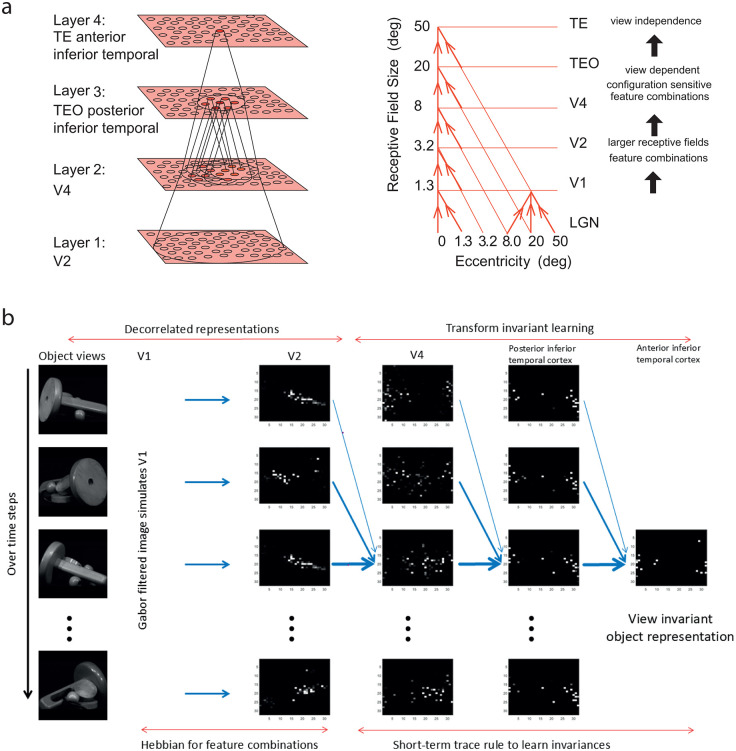
Architecture of VisNet and the visual cortex for transform-invariant object and face identification. a. Convergence in the visual system. Right – convergence in the ventral stream cortical hierarchy for object recognition. LGN, lateral geniculate nucleus; V1, visual cortex area V1; TEO, posterior inferior temporal cortex; TE, anterior inferior temporal cortex (IT). Left – convergence as implemented in VisNet, the model of invariant visual object recognition described here. Convergence through the hierarchical feedforward network is designed to provide Layer 4 neurons with information from across the entire input retina, by providing an increase of receptive field size of 2.5 times at each stage. Layer 1 of the VisNet model corresponds to V2 in the brain, and Layer 4 to the anterior inferior temporal visual cortex (TE). In this paper ‘Layer’ with a capital L indicates a Layer of a neuronal network which may correspond to a brain region as here. This is distinct from the 6 architectonic layers in neocortex, designated here with a small letter l in ‘layer’. **b: Computations performed by VisNet3.** Images are filtered with Gabor filters with four spatial frequencies, four orientations, and positive or negative to simulate V1 neuronal activity. V1 connects to V2 using a competitive net to learn feature combinations. V2 connects to V4, which connects to posterior inferior temporal cortex, which connects to anterior temporal cortex using a competitive net at each stage trained with a short-term memory rule to learn the transforms of an object that occur together close in time. By the last stage, anterior inferior temporal cortex, one set of neurons responds to every transform of a given object, and to no transforms of different objects. The panels show the firing rates in each layer of a 32x32 neuron VisNet3 to each transform of one object, where white is the maximum firing rate in the rate simulation, and black is zero. A sparse distributed representation is evident, as in the neocortex.

Rolls then investigated how these remarkable neurons might be generated in the cortical regions from V1 to the inferior temporal visual cortex, and proposed that all these invariances might be learned due to the statistics of the natural visual world, in which several transforms of a particular object or face typically occur close together in time [39]. Rolls proposed that this type of learning from the natural statistics of the visual world could be performed using slow learning with an associative learning rule with a short-term memory trace lasting 1–3 s operating in a feature combination hierarchical network of the type illustrated in Fig 1 [39]. (A trace learning rule had been suggested for translation invariance in a one-layer net by Foldiak [40]). This was tested in the four layer network illustrated in Fig 1a [41], VisNet. The input to Layer 1 comes from a simulation of V1 using Gabor filters [42]. VisNet simulates how the receptive field sizes of inferior temporal cortex neurons shrink to about the size of a single object in complex natural scenes [11,43,44], which helps to provide clear information that can be transmitted from the inferior temporal cortex to the connected memory systems about the object or face being fixated [9]. VisNet can also operate using a saliency map to locate objects in complex natural scenes, and then identify them [45]. Many different versions of the local learning rule involving just pre-synaptic and post-synaptic terms (i.e., without any backpropagation of error used in artificial deep learning networks, but including error correction and temporal difference learning) have been developed for VisNet [46–49]. Trace rule learning is an example of slow learning, has been adopted by others [50–52], and has many potential applications [49,53,54].

Key issues now need to be investigated, and provide the aims of the research described here, which all aim at enhancing biological plausibility. The first aim is to develop the synaptic modification rule used in VisNet to make it more biologically plausible. At present, in order to ensure that all neurons are equally available for learning new feature combinations and compete on an equal basis, the length of the synaptic weight vector of each neuron is normalized to 1 after every synaptic update [46–49], following investigations in competitive networks [9,55,56]. Here we investigate use in VisNet3 of a learning rule that allows for weights to decrease depending on their strength, consistent with the empirical observation that long-term synaptic depression is easier to obtain if the synaptic weights are already strong. The learning rule provides not only for a Hebbian associative increase in synaptic weight if the pre-synaptic and post-synaptic terms are both high, but also for heterosynaptic long-term depression in which a synapse decreases in strength if the post-synaptic activation is high, but the presynaptic firing is low relative to the strength of that synaptic weight, as set out in Equation 5 (see [9] Equations B.32 and B.33). We also test in VisNet3 use of the Oja rule [57], which is more complicated and perhaps less biologically plausible than what has just been described (see Equation 6), but can normalize the length of the synaptic weight vector on each neuron [56,57].

The second aim is to investigate whether having an upper limit on the strength (weight) of each synapse, which seems very biologically plausible and indeed likely, might in fact have computational advantages by helping more distributed synaptic weights to form on each neuron. We hypothesize that in a network such as VisNet3 this could help to limit the effect of some features present in different exemplars in the training set such as rectangular shapes that would with an unbounded Hebbian synaptic association rule lead to continuing climbing of some weights relative to others, and hence to difficulty in discriminating between such objects. We further hypothesize that the self-limiting synaptic rule shown in Equation 5 may help with this computational issue.

The third aim is to investigate whether removing the lower firing rates produced by the sigmoid activation function from the learning may help the operation of the network, especially in large versions of the network. This may have an effect like that of the voltage-dependent NMDA non-linearity in the associative learning rule for neurons [9], by allowing learning only for the most strongly activated neurons in the network.

Code for a new version of VisNet, VisNet3, is made available with this paper to incorporate the options described in aims 1–3, to make them very clear, and to enable experimentation with these optional extra features.

The fourth aim is to test the operation of the network at much larger scale than the 32x32 neurons in each of 4 layers of the network used in the standard model version of VisNet [49], in order to test how the model scales up, and to provide a potential basis for the model to be incorporated into an integrate-and-fire model of the human brain [58].

The fifth aim is (mainly in the Discussion) to compare the present approach to a biologically plausible brain-like network for invariant visual object recognition with less biologically plausible models that utilize backpropagation of error learning in deep networks [59–62] and lateral propagation of synaptic weights as in deep convolution networks [61,63].

## 2. Methods: Unsupervised slow learning of transform-invariant representations in a model of the ventral visual system, VisNet3

### 2.1. The architecture of VisNet3

The hierarchical organization after V1 of VisNet via V2 and V4 to the posterior inferior temporal visual cortex and then anterior temporal visual cortex with convergence from stage to stage and competitive learning is a way to set up neurons with large receptive fields that could become tuned to feature combinations that represent objects, and to produce transform-invariant representations (Fig 1a) [9,39,48,49]. VisNet is a feature hierarchy network (described in detail in S1 Appendix and elsewhere [9,64]), that emulates to some extent the sparse distributed and transform-invariant encoding that is found for objects and faces in the ventral visual system [9,15]. The hierarchical organization is important for brain systems to learn about the natural world, because it means that a single neuron need receive only a limited number (~10,000) of inputs from the previous stage (Fig 1a). Important aspects of the design to make it biologically plausible is that the whole problem is solved in a network with only 4 Layers; that a local synaptic learning rule is used; that the computation is feedforward, with no feedback of errors or anything similar required for learning; that there is no supervision of the training by for example separate teachers for each neuron in the output Layer; and that the natural statistics provided by objects as they transform in the world are used to learn transform-invariant representations.

In more detail, VisNet is a rate model that consists of a series of feedforward hierarchically connected competitive networks with convergence from Layer to Layer, with four Layers, as illustrated in Fig 1a and 1b and as described in detail in the S1 Appendix. The connections to a neuron in one Layer come from a confined and topologically related region of the preceding Layer. These connections to a neuron in one Layer come from a small region of the preceding Layer using a Gaussian distribution of connection probabilities defined by the radius that will contain approximately 67% of the connections from the preceding Layer. Table 1 shows this radius for each Layer of a network with 32 × 32 neurons per Layer, with each neuron receiving 200 synaptic connections from the neurons in the preceding Layer. The radii are set so that neurons in the fourth Layer of VisNet are able to be influenced by inputs from a stimulus at any location in Layer 1 [48]. Details with parameters used in a small network are provided in Table A in S1 Appendix, and in a larger 256x256 VisNet3 with up to 1000 synapses per neuron in Table B in S1 Appendix. The activation of a neuron is calculated as the synaptically weighted sum of the rate inputs it receives from the preceding Layer, i.e., as a dot or inner product between the input rates and the synaptic weights [9,46,48,65]. The activations are converted into rates with a sigmoid or threshold-linear activation function, with the sparseness of the representation in a Layer set as described in Section 2.3 and in more detail in S1 Appendix.

**Table 1:** VisNet3 default parameters for the Matlab small version (see also Table A in S1 Appendix). Dimensions shows the number of neurons in each of the 4 Layers. # Connections shows the number of synaptic connections onto each neuron. Radius shows the radius of the connectivity from the previous Layer of a single neuron (see text). This is for the small tutorial version of VisNet3 written in Matlab and made available with this paper. This tutorial version of VisNet3 can be scaled up to at least 256x256 neurons per Layer, and 1000 synaptic connections to each neuron.

	Dimensions	# Connections	Radius
Layer 4	32x32	200	7
Layer 3	32x32	200	7
Layer 2	32x32	200	7
Layer 1	32x32	340	15
Input layer	256x256x32	–	–

### 2.2. The short-term memory trace learning rule used in VisNet

A key part of the proposal for VisNet is learning that uses a short-term memory trace for previous neuronal activity, so that the neurons could learn to respond to different transforms of an object, which in the real world typically occur close together in time [39]. A similar principle had been proposed for translation invariance in a one-layer network [40], but Rolls extended this to all types of invariance, and outlined how this could be set up in a hierarchical model of processing in the ventral cortical visual stream to the inferior temporal visual cortex [39]. The full model was built [41,66], which is known as VisNet [48], and a reduced version of which in Matlab is available with *Brain Computations and Connectivity* [9]. The trace learning rule is biologically plausible, and could involve processes such as the long time constant of NMDA receptors, or local cortical attractor network operations, which do keep cortical neurons firing for a few hundred ms after a stimulus [9,67,68].

The short-term memory trace that enables inputs occurring close together in time, as they would in the natural world, to become associated is implemented in the hierarchical competitive network [9,48] model by using associative synaptic modification with a small change that allows the postsynaptic term to remain active for short periods in the order of 100 ms or more. The short-term memory trace update learning rule that we have used has the following form [9,48]:


$$\delta w_{j}=\alpha {\bar{y}}^{\tau }x_{j}$$ (1)

where


$${\bar{y}}^{\tau }=(1-\eta )y^{\tau }+\eta {\bar{y}}^{\tau -1}$$ (2)

and

$x_{j}$ is the $j^{th}$ input to the neuron;

y is the output from the neuron;

${\bar{y}}^{\tau }$: is the Trace value of the output of the neuron at time step τ;

α is the learning rate;

$w_{j}$ is the synaptic weight between $j^{th}$ input and the neuron;

η is the trace update proportion, with 0 meaning no trace, just associative learning. The optimal value varies with the number of transforms of each object, and is typically 0.8. During training, the firing yτ of a neuron in a layer to the presentation of a transform of an object is computed as described in this Methods section and in the Supplementary Material, and the short-term memory trace ${\bar{y}}^{\tau }$ is then updated as shown in Equation 2. The synaptic weights are then updated after every presentation of a view of an object as shown in Equation 1 using this short-term memory trace ${\bar{y}}^{\tau }$.

Many variations of this learning rule have been explored [46,47]. It was demonstrated that a modified Hebbian rule that incorporates a trace of previous activity but no contribution from the current activity can offer substantially improved performance for trace-rule learning [46]. We then showed how this rule can be related to error correction rules, and explored a number of error correction rules that can be applied to and can produce better invariant pattern recognition learning than the simple rule shown in Equation 1. An explicit relationship to temporal difference learning was then demonstrated, and from that further learning rules related to temporal difference learning were developed [47]. However, because errors needed to be calculated for these learning rules, and that may not be biologically plausible, the default rule used in research with VisNet including that described here is the simple Hebbian type of associative learning rue shown in Equation 1, as that is biologically plausible. The general form of the rule for computational purposes can be as shown in Equation (1), but the actual mechanism in the brain might utilize a slow synaptic eligibility trace such as provided by the NMDA receptors with their long time constant, as well as a tendency for neuronal firing to continue due to local attractor networks [9,48].

During training, all transforms of one object are presented in random sequence so that the trace rule can help learning that all of these are transforms of the same object because they occur close together in time; then all transforms of another object are shown; etc. This emulates how one object at a time is typically looked at in the natural visual world.

As described previously [46], the learning is somewhat better if the trace term ${\bar{y}}^{\tau }$ used in Equation (1) is from the previous timestep only without a contribution from the present transform (e.g., view) about which no learning may yet have taken place, and that is incorporated by default in the VisNet Matlab code that is made available, and was used in the simulations described here. That can be described as using a trace ${\overset{―}{y}}^{\tau -\text{1}}$. (The reason that this is useful is that the synapses are updated based on the previous views seen recently of an object, without including any firing produced by the current view of the object, which might not have been seen before or learned about well yet.)

Layer 1 of VisNet is trained with a purely associative learning rule with no short-term memory trace, to enable feature combination neurons to be formed that represent the relative spatial locations of the features before any invariance learning starts in Layer 2. This helps to solve the feature binding problem, as described below and elsewhere [6,9,41,48]. By feature binding, we mean forming a neuron that responds for example to a vertical and horizontal bar in a given spatial arrangement so that for example an ‘L’ feature is formed. It is essential to perform some feature binding before transform invariance is computed, for otherwise all the shape descriptors necessary to describe an object would be jumbled up with respect to each other, and no particular shape could be specified. The operation of feature hierarchy networks is described in detail elsewhere [6,9].

### 2.3. Competition and mutual inhibition in VisNet

Each layer of VisNet operates as a form of competitive network, in which different neurons learn to represent different categories of inputs, as described next and in more detail elsewhere [9]. In a competitive network, an input vector of firing rates is applied to the randomly initialised synaptic weight vectors on each neuron, some neurons are activated more than others, and inhibition between the neurons leads to a small subset of neurons remaining firing after the competitive interaction implemented by the inhibitory neurons. The synaptic weights of each neuron then learn by associative learning based on the presynaptic and postsynaptic firing rates. In a competitive network [9], mutual inhibition is required between the neurons within each Layer, so that for any one input stimulus only a proportion of neurons is active. This selection is performed in VisNet3 by setting a threshold such that only a set of the most activated neurons corresponding to the sparseness required is selected to be firing within a layer. For example if the sparseness *a* defined in Equation 3 is 0.01, then 1% of the neurons would be selected to be firing within a layer for that stimulus. This is thus not a winner-take-all network, but has graded firing rates of neurons with a sparseness that defines the proportion of neurons that are firing after the competition. These details, and how lateral inhibition between the neurons within a layer is implemented, are described in Section 1.5 of the Supplementary Material. Exactly how the particular sparseness of the representations that are found in cortical regions [15] are produced may best be investigated in integrate-and fire networks, and will depend on inhibitory neurons and the excitatory-inhibitory balance [6,69].

We emphasise that each Layer in VisNet and VisNet3 is not a competitive network with a single winner, but has multiple neurons left firing with different firing rates in a sparse distributed representation, for that is how information is encoded in the cerebral cortex [6,9,15] (see Fig 1b). The activation of the neurons in a Layer is first calculated by the dot product of the synaptic weights of a neuron and the rates of the neurons in the preceding Layer to which it is connected by the synaptic weights. Then the activations are converted into rates using a sigmoid or threshold linear activation function, and the threshold for the activation function is set so that the sparseness across the neurons of the rates becomes a value specified by a sparseness parameter a that is typically 0.01, where sparseness is defined as


$$a=\frac{{\left(\sum_{i}y_{i}/n\right)}^{2}}{\sum_{i}y_{i}^{2}/n}$$ (3)

where n is the number of neurons in the Layer, and $y_{i}$ is the firing rate of the i th neuron in a Layer. Setting the sparseness in this way implements a form of competition within the network, in that only the neurons with the highest activations have rates greater than zero after the sparseness has been set as specified. This measure of sparseness is one that is useful in the quantitative analysis of the capacity of neuronal networks [6,9,64,70–72], and in neurophysiological measures of neuronal representations in the brain [6,9,15,64,73,74]. If the neurons have binary rates, the sparseness is the proportion of neurons that is active for any one stimulus.

In VisNet, typically a sigmoid activation function is used


$$y=\frac{\text{1}}{\text{1}+e^{-\text{2}\beta (r-\alpha )}}\;$$ (4)

where *r* is the activation of the neuron, *y* is the firing rate produced by the activation function, β is the slope or gain, and α is the threshold or bias of the activation function, which in VisNet3 reflects the sparseness parameter *a*. The sigmoid function bounds the firing rate between 0 and 1. In VisNet3 when we train large versions of the net, we may increase the slope β to minimize the contribution of low firing rate neurons to what is learned by the synapses in the network. This may help to increase the number of objects that can be learned in large networks, beyond what might be produced just by decreasing the sparseness.

It is emphasised that a competitive network, which provides the basis for the learning in each layer of VisNet, is a feedforward unsupervised network [6,9]. An input vector is applied to the neurons in the network, and their activations are calculated. The neurons with the highest activations for the current input vector of firing rates are selected to be firing according to the sparseness parameter *a* that is specified, and a sigmoid activation function is used. That process is repeated for the set of input firing rate vectors that are to be categorised by the competitive network. There are no recurrent collaterals in the network, so that this is not an attractor network. There are no error correcting backprojections or top-down influences. A competitive network is thus an unsupervised feedforward network, with the simple architecture and operation described further and illustrated fully elsewhere [6,9].

### 2.4. Synaptic weight normalisation or scaling in VisNet3

In a competitive network, it is important that all neurons compete on an equal basis, so that different neurons learn to respond to different inputs, and similar inputs are allocated to the same neuron, so that categorisation is performed usefully [9]. The usual way in which this is implemented in a competitive net is that after learning with a Hebbian associative rule or one with a short-term memory trace in the post-synaptic term *y* such as that in Equation 1, the length of the vector of synaptic weights on a neuron is set to one [9,55,56]. Given that a Hebbian rule will always increase synaptic weights if the presynaptic and postsynaptic firing rates are greater than 0, the synaptic modification will increase some synaptic weights. The weight normalisation (setting the sum of the squares of the weights = 1) will then decrease the weights usefully [9,56]. That is what is implemented in VisNet, by dividing the synaptic weight vector on a neuron by the length of its synaptic weight vector after its synapses have received an update [48,49].

However, setting the length of the synaptic weight vector on each neuron after every synaptic weight update is not very biologically plausible. So for VisNet3 we introduce an alternative method of allowing each neuron to compete on an equal basis by using in VisNet3 a learning rule that allows synaptic weights to decrease in value if they are on a strongly activated neuron, and the current weight is larger than the presynaptic term. This provides for heterosynaptic long-term depression, which may be easier to obtain if the synaptic weights are already high [75], in addition to long-term potentiation. The rule we introduce for VisNet3 is


$$\delta w_{j}=\alpha y\;(x_{j}-w_{j})$$ (5)

This rule was used in different applications previously [56,76,77], and has been termed the ‘standard competitive net learning rule’ [56]. Here, we compare the operation of VisNet3 using this ‘standard competitive network rule’ shown in Equation 5, with the weight normalisation used in VisNet [48,49], and with the Oja rule [56,57,78] shown in Equation 6


$$\delta w_{j}=\alpha y\;\left(x_{j}-yw_{j}\right)$$ (6)

which though somewhat similar to what is shown in Equation 5 can normalise the synaptic weight vector and is we suggest less biologically plausible than the rule shown in Equation 5. (The Oja rule may be less biologically plausible as it was designed to make the vector length of the synaptic weights scale to the same value, and to do that requires a quadratic use of *y* as shown above, and the evaluation of that quadratic term may not be biologically plausible.) It will be shown in the results that training VisNet3 with Equation 5 produces somewhat better performance than with Equation 6 if non-binary firing rates are used, and much better than that achieved with the associative learning and weight normalisation used in previous versions of VisNet. The default learning rule used in VisNet3 is that shown in Equation 5, but using ${\bar{y}}^{\tau }$ instead of *y*, and replaces what is shown in Equation 1 that was used in previous versions of VisNet. We note that if the sigmoid parameter β is large, the firing rates will be binary, and with the maximum firing rate of 1 produced by the sigmoid activation function, then Equation 6 for the Oja rule reduces to Equation 5 for the standard competitive net learning rule.

### 2.5. Limiting the maximum synaptic weight on a neuron

With normal Hebbian learning using a rule like that shown in Equation 1 (but without a short-term memory trace on the post-synaptic term *y*) some synaptic weights might continue to increase to high values, especially if some features are present in different objects. It seems biologically implausible that synaptic weights could grow without bound, so we have investigated limiting the maximum value that a synaptic weight on a neuron can reach (set with MAX_WEIGHT in the Matlab code for VisNet3). (It seems implausible that synaptic weights could grow without bound, in that if we train for example 10,000 memories into an associative network using an associative Hebbian learning rule, the strongest weight could be 10,000 times the strength of a weak weight, and that range or precision seems implausible. In fact, when we do the capacity calculations, we find that each synapse in an associative network trained to capacity would only store about 0.2-0.3 bits of information [6,9,70], so high values and high precision of synapses seem not only implausible, but also unnecessary [6,9]). We in fact propose that allowing the synaptic weights to saturate at a maximum value could be beneficial, by encouraging neurons not to rely on a few strong synaptic weights from high-firing inputs to the neuron, but to grow weights from a number of inputs, in order to increase the sampling of information from the preceding layer by producing a more distributed synaptic weight representation for what is learned by each neuron. We show in the simulations presented in the Results that this can be useful in at least large network versions of VisNet3. This process is typically combined with other methods to scale the weights on a neuron, such as the procedure implemented in Equation 5. In practice, the algorithm is to update the synaptic weights during learning for every view of every object as shown in Equation 5, and at the same time to ensure that the maximum synaptic weight on a neuron is clipped to the value of MAX_WEIGHTS, typically 0.06 when it is set. Given that the maximum firing rate *y* of a neuron is 1.0, and that the maximum presynaptic firing rate *x*_*j*_ is 1.0, the maximum that a weight could ever be according to Equation 5 is 1.0 without any maximum weight clipping, and in small versions of VisNet might reach 0.8 as illustrated in Fig 3b.

### 2.6. The inputs to VisNet are provided by V1-like neurons produced by Gabor filtering of input images

The inputs to VisNet are computed to have elongated receptive fields of the type found in the primary visual cortex V1, in order to allow comparison of the neurons in VisNet at different stages to those in the brain. The Gabor filters [79] have four spatial frequencies, four orientations, and positive or negative as described in the Supplementary Material. The Layer 1 neurons are connected to these with radii as described above and in Table 1, and with the number of connections to each frequency scaled according to the spatial frequency, as described in detail elsewhere [9,48,65] and as shown in Table A in S1 Appendix.

### 2.7. The capacity of VisNet

Several factors that make a useful contribution to the number of objects that can be recognized by VisNet have been described above. These factors include the use of sparse distributed representations, and the reuse of intermediate-Layer neurons as components of different objects represented at the final Layer [48]. But how VisNet would scale up to provide a model of human visual object representations is a topic of interest. VisNet in quite a small form of 32x32 neurons in each of 4 Layers, and 200 synapses on to each neuron from the preceding Layer, is small compared to what is found in the neocortex. Cortical pyramidal cells often have in the order of 20,000 synapses per neuron, with perhaps 10,000 devoted to recurrent collateral inputs, perhaps 5,000 synapses to feedforward inputs that could be used for competitive learning, and perhaps 5,000 to backprojections ending in layer 1 [6,9,64]. The number of neurons in such a cortical module might be in the order of 100,000 [6,9,64]. Each such module would occupy a region of the cortical mantle with an area of a few mm^2^. An important property is that this connectivity is diluted, with the dilution in the order of perhaps 0.1, and that could help with capacity, as each neuron potentially receives a different combination of the afferents from the preceding cortical area. The ventral (in fact ventrolateral [37,80]) cortical visual system could have tens to hundreds of such modules [6,9,64].

With these factors in mind, it is difficult to know whether VisNet would scale up sufficiently to account for primate/ human visual object recognition. To investigate how this architecture may scale up, we describe for VisNet3 a larger network with 256 × 256 neurons in each layer, and up to 1,000 synapses per neuron, using up to 800 objects each with up to 9 views from the Amsterdam Library of Images (ALOI) [81]. Each ALOI image was carefully sized to occupy the full 256x256 image space and centred before the GABOR filtering, and was used in grayscale. In the capacity simulations that are reported here, VisNet3 was trained with 9 views of ALOI objects, starting from the beginning of the ALOI object list, and testing different numbers of objects until the performance measured with the object selectivity measure described next decreased. The testing was with the same 9 views, so this can be described as within-set transform invariance testing. The testing of how the number of synapses per neuron influenced the capacity was performed as follows. One set of parameters was chosen for these investigations, and the only parameter that was changed was the number of synapses per neuron. The parameters selected were those known to produce good performance of the whole network with up to 1000 synapses per neuron, are shown in Table B in S1 Appendix, and include a sparseness of the firing in each layer of 0.0025, a β for the slope of the activation function of 100, and a value for the maximum synaptic weights of 0.06 for layers 1–3 where this parameter can be useful. Operation with each value of the number of synapses per neuron being tested was run 10 times with different random seeds for the network architecture, and the standard deviations of the object selectivity across these 10 simulations were in the range 0.001-0.005, so were negligible in relation to the object selectivity criterion of 0.6.

**Fig 2:**
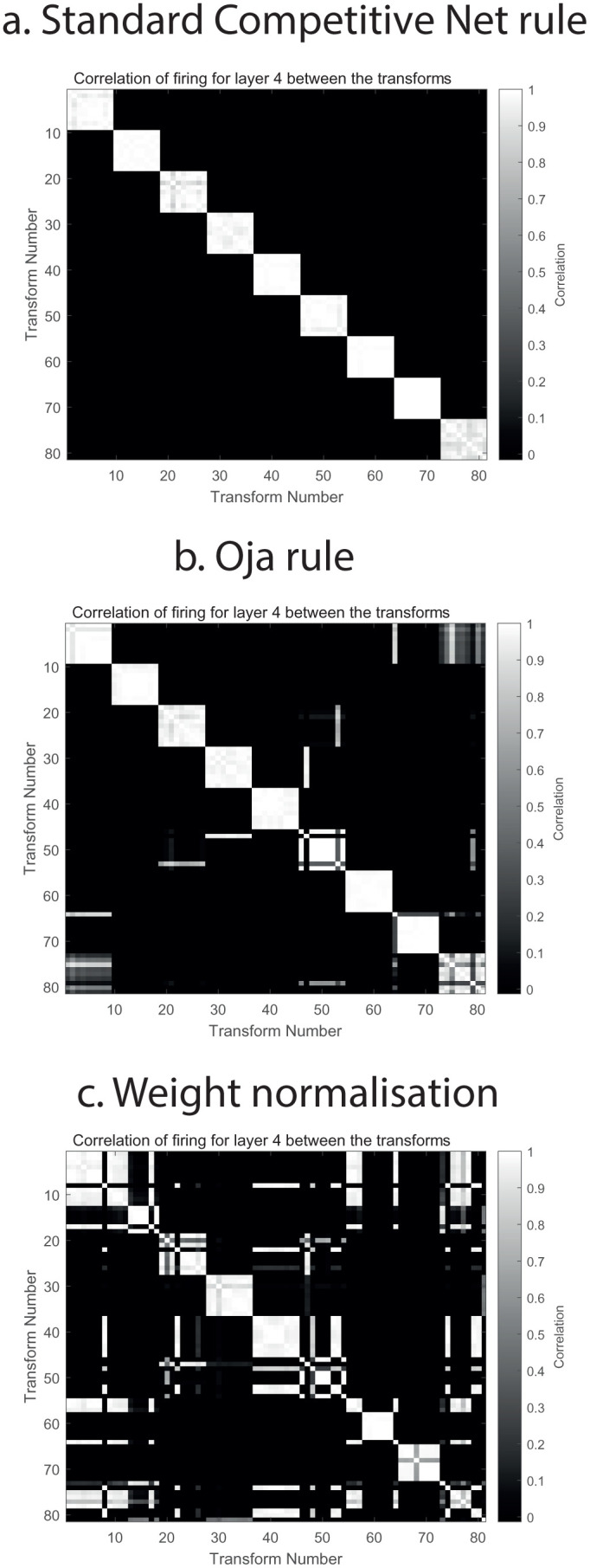
Comparison of learning rules for VisNet3. VisNet3 was trained on 9 objects each with 9 views with the parameters shown in Table A in S1 Appendix. The correlations shown in all Figures are the correlations between the firing rates of all the neurons calculated as described in the text in the specified layer of VisNet that are produced by different views of an object. In this case, 9 objects were trained and tested, each with 9 views. (a) shows the performance when trained on the ‘standard competitive net rule’ shown in Equation 5. The first 9 transforms are the different views of object 9. The second 9 transforms are the 9 views of object 2. The 9 views of object 1 are highly correlated with each other, and not with any view of any other object, etc. The view invariant object representation is thus almost perfect, with an object selectivity = 0.98. (b) shows the performance when trained with the Oja rule shown in Equation 6. The performance was a little less good with an object selectivity = 0.78: views of some objects were correlated with some views of other objects. (c) shows the performance when trained with a Hebbian associative rule like that shown in Equation 1, followed after each synaptic update with normalization of the length of the synaptic weight vector. The performance was less good with an object selectivity of 0.39.

The invariant representation of each object after training was measured by an object selectivity measure derived from the correlation matrices between the firing rates in layer 4 to the set of all views of all objects, illustrated for example in Fig 2. The object selectivity measure was the sum of the correlations between the firing to the different transforms of each object, divided by (the sum of the correlations between the firing to the different transforms of each object for perfect representations + the sum of the correlations between the firing for each object to the firing for all transforms of all other objects). The maximum value for perfect view invariance for each object and no response to any other object is 1.0, and the minimal value is 0. With each object represented by 9 views, perfect performance would be shown by high correlations between the 9 different views of any object, and no correlation with any view of any other object, as illustrated in Fig 2a. Less good performance, with the corresponding object selectivity measures, is illustrated in Fig 2b and 2c.

## 3. Results

### 3.1. Learning rules with different weight scaling/normalization

The Matlab small version of VisNet3 with the parameters shown in Table A in S1 Appendix was trained with the three different rules described in section 2.4. Use of the ‘standard competitive net rule’ shown in Equation 5 produced the correlations in Layer 4 between the 9 objects each trained with 9 views shown in Fig 2a, with an object selectivity = 0.98. Fig 2b shows the performance when trained with the Oja rule shown in Equation 6. The performance was a little less good with an object selectivity = 0.78: views of some objects were correlated with some views of other objects. These two synaptic update rules produced generally comparably good performance in VisNet3. We prefer use of the standard competitive net rule of Equation 5 in VisNet3 because it is more biologically plausible than the Oja rule shown in Equation 6. Statistical analyses with new random seeds to construct the networks but the same parameters as used for Fig 2 showed that across 20 runs the mean Object Selectivity for the standard competitive network learning rule of Equation 5 was 0.92 (SD = 0.05), for the Oja rule in Equation 6 was 0.77 (SD = 0.06), with t = 8.87, df = 38, p < 10^-10^.

Fig 2c shows the performance when trained with a Hebbian associative rule like that shown in Equation 1, followed after each synaptic update with normalization of the length of the synaptic weight vector on each neuron. The performance was poorer with an object selectivity of 0.39. (Across 20 runs the mean Object Selectivity for the Oja rule in Equation 6 was 0.77 (SD = 0.06), for the weight vector length normalization was 0.44 (SD = 0.06), with t = 19.6, df = 38, p < 10^-10^.) A reason for the poorer performance of the synaptic weight normalization used with associative increases of synaptic weight than the standard competitive net rule (Equation 5) is that the latter specifically decreases strong synaptic weights if the postsynaptic term *y* is high, and the presynaptic term *x*_*j*_ is low, thereby potentially reducing effects of other stimuli on that neuron. In contrast, with weight normalization all synaptic weights are scaled down equally after the synaptic update, so that process builds less selective neurons.

### 3.2. Clipping the maximum strength of synapses

**Fig 3:**
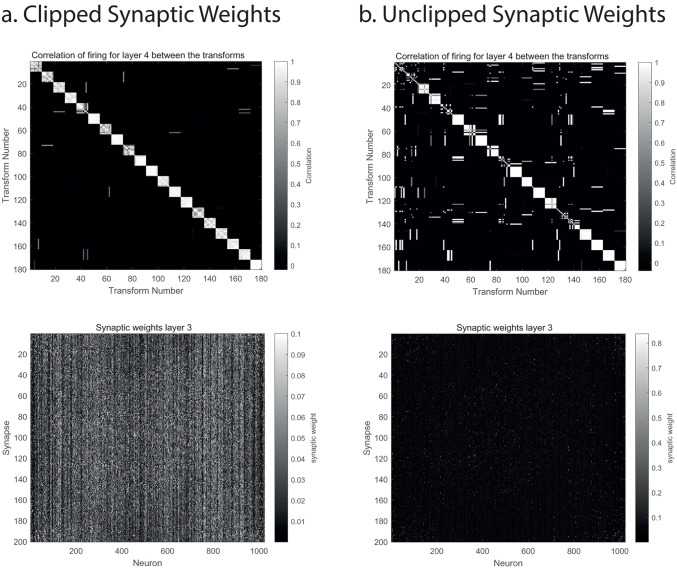
Clipping the synaptic weights at a maximum value can increase performance. a. During training, the maximum value of the synaptic weights in layers 1-3 was clipped at 0.1. Above: the correlation matrix between the 20 objects each trained with 9 views. The Object selectivity was 0.86. Below, the synaptic weights in Layer 3 of the network, illustrating that many synapses on each neuron could contribute to its activation. In the 32 × 32 neuron network, the 200 synaptic weights on each of the 1024 neurons are shown as the vector or column of values for each neuron. b. With no clipping. Above, the Object selectivity was 0.49. Below, relatively few but strong synaptic weights on each neuron may be visible, and these few strong synaptic weights make a large contribution to the activation of the neurons. The full set of parameters used are provided in Table A in S1 Appendix.

Fig 3a shows that clipping (limiting or saturating) the synaptic weights to a maximal value during learning can increase the performance of VisNet3, with an Object selectivity of 0.82 compared to the unclipped condition shown in Fig 3b in which the Object selectivity was 0.66. The parameters for these simulations are shown in Table A in S1 Appendix.

When operating with associative learning rules such as that shown in Equation 5, some synapses can become quite strong, close to 1.0 given that the maximum presynaptic rate is 1.0, and the maximum post-synaptic rate is 1.0. Fig 3b shows that when operating in the standard way, without weight clipping, some of the synaptic weights can reach values of more than 0.7, and there are relatively few such strong synaptic weights on any one neuron. In that situation, if only one presynaptic neuron was active for each image being presented, and only one post-synaptic neuron was active, the whole network would operate somewhat like a look-up table. VisNet ensures that this is not the operating regime, by typically using sparseness values of 0.01 or larger for the firing rate representations in each layer, to ensure that a number of neurons are active when any one image is being presented, and thus operates in the regime of sparse distributed representations, somewhat similar to the firing of neurons in inferior temporal visual cortical regions to faces and objects [9,15].

However, when the maximum value of a synaptic weight is clipped during training, to for example 0.1, it is shown in Fig 3a (lower) that many synaptic weights on each neuron (each neuron’s weights are a column in the Figure) become almost as large as the maximum weight on a neuron. A result is that each neuron uses more of its synaptic weights to represent the different inputs to which it can respond. In VisNet3, this helps the whole network to learn how to map the many different inputs that characterize any one object or feature into the same output neurons, and thereby improves the performance.

We hypothesize that this synaptic clipping way of enhancing the performance of some neuronal networks, such as VisNet3, by enhancing a broad distribution of synaptic weights on any one neuron, is different from altering the sparseness of the firing rate representations (Equation 4). A sparse distributed firing rate representation is advantageous for enabling some generalization to similar stimuli or events, when a similar though not identical set of neurons may be active, because of the distributed nature of the representation [9,15]. At the same time, the sparse property of the representation enables a high storage capacity, in both autoassociation attractor networks [9,70,71] and in pattern association networks [9,72]. But having separate control over the distributed nature of synaptic strengths on a neuron could enable different input firings produced by for example different views of an object to become associated onto the same neuron. In other words, weight clipping or saturation can enable neurons to learn about two completely different input patterns. This applies naturally in a network with the trace rule for invariant representations, for the trace of previous neuronal activity could enable a neuron to learn about two orthogonal input patterns of neuronal activity, as seen for example on recent but not the current view of an object. Another situation in which this could be useful in when there is a teacher forcing each neuron to respond, as in a pattern association network, in which two orthogonal input patterns could be learned onto the same output neuron [9,72].

We note that in practice, the use of clipping the synaptic weights at a maximum value is especially useful in a heavily loaded network.

Overall, we propose here that synaptic weight clipping is an interesting principle of cortical computation that can have advantages in some neuronal networks, and for example in VisNet3 can increase the number of objects that can be learned with transform-invariant representations.

### 3.3. Performance of a scaled up version of VisNet3

VisNet3 was scaled up to 256 × 256 neurons in each layer, and up to 1000 synapses per neuron, to investigate how its performance scales up, and what factors influence the capacity. (There are up to 262,144,000 synapses in this 4-layer network.) The training stimuli used were up to 800 objects each with 9 views spaced 40° apart from the ALOI dataset [81]. (The value of 40° apart was used because of prior work that had shown that VisNet performs well when trained with object views separated by about this amount [45,49]. Moreover, such a set of views shows all the visual features present in most objects.) The key parameters used for the training are shown in Table B in S1 Appendix.

**Fig 4:**
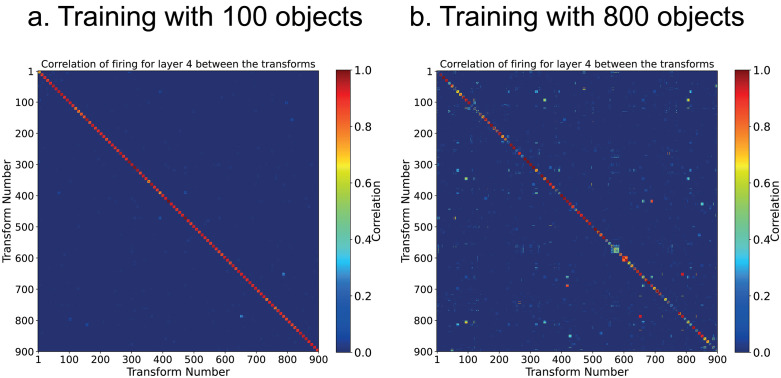
Performance of a larger version of VisNet3 with 256*256 neurons in each layer, and up to 1000 synapses per neuron. (a) Performance when trained on 100 Amsterdam Library of Images objects each with 9 views, for which the Object Selectivity was 0.85 with 1000 synapses per neuron and MaxWeight = 0.06 for Layers 1-3. (b) Performance when trained on 800 ALOI objects each with 9 views, for which the Object Selectivity was 0.50. For (b), the correlations for just the first 100 of the 800 objects are illustrated, for readability, and the Object Selectivity was measured for these 100 objects, as some of the ALOI objects later in the series are more confusable, and to allow direct comparison with the object selectivity in (a) in which 100 objects were trained. For almost all objects, along the diagonal there is a small block of 9x9 correlations for the different views of each object, with correlations off the diagonal for only a few objects that were somewhat similar in form and confusable. There were 9 transforms of each object. The training parameters are shown in Table B in S1 Appendix.

Fig 4a shows the correlation matrix between objects when trained on 100 objects for which the object selectivity was 0.85, and in Fig 4b when trained on 800 objects for which the object selectivity was 0.50.

The key parameters shown in Table B in S1 Appendix enabling this good performance were as follows. The large number of synapses per neuron were especially important, as shown by systematic experiments in which these were altered with results described in section 3.5. So was the sparseness of the representation (Table B in S1 Appendix), with more sparse values being useful when a large net was heavily loaded with objects. Although some of these values seem quite sparse (e.g., 0.0025), we note that in a cortical module of 260,000 neurons, this would mean that the firing of 640 neurons was contributing to the learning at any one time. Moreover, what is stored in the synaptic weights may reflect only the neurons with higher firing rates, given the voltage-dependent non-linearity of NMDA receptors, so the sparseness of the representation that is learned may be lower (more sparse) than that indicated by the whole firing rate distribution [6,9,15].

The use of the ‘standard competitive net learning rule’ shown in Equation 5 produced much better performance than weight normalisation after Hebbian associative learning, for the reason described above. All the results presented in this section were with this ‘standard competitive net learning rule’ shown in Equation 5. The use of MAX_WEIGHTS was also important in obtaining good performance.

The fact that performance of a competitive network becomes poorer with increased requirements for the number of categories to be produced is not surprising, with one limit being set by the number of output neurons and the sparseness of that output representation (with a lower value for the sparseness increasing capacity up to a limit set by the number of output neurons); and the number of input synapses and the sparseness of the inputs also being a factor that helps to determine how well training firing rate vectors for the inputs can be separated, as considered elsewhere [6,9].

### 3.4. An activation function that minimizes low firing rates in a neuronal network, and that may thereby enhance learning; and the effects of the sparseness of the representation

The activation function typically used in VisNet is a sigmoid activation function (Equation 4), as set out in section 2.3. In the large networks investigated in section 3.5, it was found that increasing the slope of the activation function β in Equation 4 from the default value of 10   to 100 or more can improve the performance of VisNet3. For example, in Fig 4a, β was set to a high value of 1,000 to emulate a binary activation function, and the object selectivity was 0.85, but with β set to the default value of 10 to emulate the graded firing rate representations found in the cerebral cortex, the object selectivity at 0.62 was lower. The effect of this is to reduce the number of neurons in a layer with low firing rates, as instead they are set to 0. This effectively means that during learning, neurons with what would have been low firing rates are excluded from any synaptic increases, because of their zero rates. This may be a way of reducing some interference between objects that are being learned. As noted in section 2.3, this may be conceptualised as setting some threshold in VisNet3 on whether a neuron shows synaptic plasticity. A different way to alter which neurons are involved in learning is to set the sparseness of the representation in a layer, set in VisNet3 by the parameter *a*.

We note that in the brain, and in an integrate-and-fire network, what is actually learned by neurons may be set by the voltage-dependent threshold for synaptic modification implemented by the properties of the NMDA receptor, as shown in Eqn B.35 of Rolls [9]. In more detail, the NMDA receptors that are involved in synaptic modification are only likely to be strongly activated at moderate to high firing rates of neurons [82]. This will result in the population of neurons that learn being somewhat more sparse than is measured by the full firing rate distribution of neurons within a brain region [6,9], and this may be an important factor in increasing the capacity of a network beyond what is calculated from the sparseness of the full firing rate distribution. In VisNet3 this could be implemented by reducing the sparseness of the firing rate representation in a layer.

### 3.5. Capacity estimates for VisNet3

**Fig 5:**
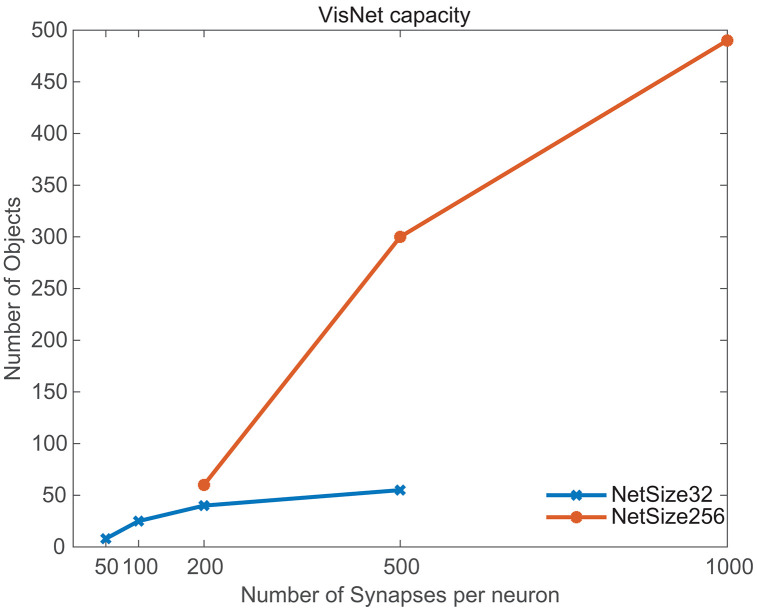
Capacity estimates for VisNet3. The number of objects that can be stored with invariant representations over 9 views for VisNet3 with 32 × 32 neurons and 256 × 256 neurons in each of 4 layers. The capacity estimates are shown as a function of the number of synapses onto each neuron. The performance criterion was an Object Selectivity of >0.6. The parameters for the simulations are shown in Tables A and B in S1 Appendix.

Fig 5 shows how the number of objects that can be learned with invariant representations over 9 views spaced 40 deg apart varies with the number of synapses per neuron, and for 2 sizes of VisNet3, with 32 × 32 neurons in each of 4 layers, and with 256 × 256 neurons in each layer. The parameters such as sparseness and MAX_WEIGHTS were optimized for each data point for Layers 2 and 3 of VisNet3 as shown in Tables A and B in S1 Appendix with it being advantageous to decrease the sparseness as the number of synapses per neuron was increased. The value of MAX_WEIGHTS for these simulations was [0.06, 0.06, 0.06, 1]. Fig 5 provides evidence that the capacity increases with the number of synapses onto each neuron, with the maximum that could be tested = 1,000, compared to the number of synapses on each neuron in higher visual cortical regions in the order of 10,000 [9,49]. The capacity may also increase with the number of neurons in the network, with the maximum that was tested = 65,536. This compares to a number of pyramidal cells in a small region of neocortex within 2 mm of approximately 260,000 (if a neuronal density of 30,000/mm^3^ is assumed [9]).

## 4. Discussion

Several key advances have been made in the research described here in increasing the biological plausibility of biologically plausible networks for invariant object and face recognition in the primate including human ventrolateral visual cortical pathway leading from V1 to V2 to V4 to posterior inferior temporal visual cortex to anterior inferior temporal visual cortex.

One key advance is use of the synaptic learning rule shown in Equation 5 which can be termed the ‘standard competitive net rule’. Before this, in VisNet [9,48,49], simple Hebbian associative increases in synaptic weight like those shown in Equation 1 based on how high the presynaptic and post-synaptic firing rates are, followed by normalization of the length of the synaptic weight vector on each neuron, was used, following an early model of competitive learning [55]. That synaptic weight normalization involved is not very biologically plausible. In contrast, the ‘standard competitive net rule’ shown in Equation 5 does not need any explicit synaptic weight normalization on each neuron, for a synaptic weight can decrease in strength if the neuron is strongly activated as shown by *y*, and if the existing synaptic weight strength is higher than the presynaptic firing rate *x*_*j*_. This is a form of heterosynaptic long-term depression, in which synapses with low presynaptic firing decrease in strength onto a postsynaptic neuron with high activity, which can be advantageous in a number of ways in neuronal networks [6,9]. In the present context, an advantage is that this can reduce the synaptic weight of inputs to a neuron that are not involved in activating the neuron, with the result that later that neuron will not be activated for inputs that may be from other objects. That is part of the mechanism for increasing the selectivity of each neuron in the network to only one or a few objects, and thereby reducing interference from other objects during later recognition. In contrast, synaptic weight normalization does not increase the selectivity of a neuron to some inputs, but merely scales down all the synaptic weights in the same proportion.

An advantage of the ‘standard competitive net rule’ shown in Equation 5 is evident in VisNet3 as illustrated in Fig 2, which shows much better object selectivity for this rule (Fig 2a) than for Hebbian learning with weight normalization (Fig 2c). The Oja rule [57] shown in Equation 6 performs in VisNet3 almost as well as the ‘standard competitive net rule’ (Equation 5), as also borne out in the large version of VisNet3 (see for example Fig 4). The somewhat less good performance of the Oja rule may be because for low postsynaptic rates *y*, any corrective decrease of a high synaptic weight *w*_*ij*_ will be less than for the standard competitive net rule in Equation 5, because the amount of decrease of the synaptic weight for the Oja rule is scaled by the low value of the rate *y* in Equation 6.

The ‘standard competitive net rule’ shown in Equation 5 has biological plausibility in that in experiments, heterosynaptic LTD may be easier to obtain after the synapses have been potentiated [75]. However, an implication of the present research is that new research would be very useful on whether what is shown in Equation 5 is found in the neocortex, given the potential computational importance of this type of synaptic weight-dependent heterosynaptic long-term depression for understanding neocortical function [6]. It is noted that this learning rule has been described before [56,76,77], but here we show how useful it is in this hierarchical model VisNet3 of the operation of the ventral visual cortical stream for object recognition. As far as we know, this is the first demonstration in a hierarchical model of cortical invariant visual object recognition that this standard competitive network learning rule (Equation 5) performs better than the classical associative learning rule (Equation 1) followed by synaptic vector length normalization [55]. We emphasize that it would be very useful to explore how its two key aspects, heterosynaptic long-term depression, and LTD that depends on the magnitude of the synaptic weight, is implemented in the cerebral cortex. A different rule was proposed that attempts to regulate the synaptic weights homeostatically using a time-averaged value for the post-synaptic firing [83], but it is not useful in the current context as it forces all neurons to learn for given inputs, which is not desirable in competitive nets, where some neurons should remain unallocated for future input patterns. The standard competitive network learning rule of Equation 5 may be helpful not only in many parts of the cerebral neocortex where new representations need to be learned [6,9,37], but also in parts of the hippocampal system such as the dentate gyrus and CA1 where new representations need to be formed [84,85].

A second advance in understanding biologically plausible neuronal networks for invariant visual object recognition is that limiting the maximum strength of synapses may not only be biologically plausible, but may have computational advantages. When used in the large version of VisNet3 described here, this could improve performance, by we propose facilitating the use of many inputs to a neuron from the preceding layer of the network, which makes it into a more distributed computational system and less of a look-up table based on a few very strong synapses, with potential computational advantages for generalization, for example to interpolated images. In particular, we propose in section 3.2 that weight clipping or saturation can enable neurons to learn about two completely different input patterns. This applies naturally in a network with the trace rule for invariant representations, for the trace of previous neuronal activity could enable a neuron to learn about two orthogonal input patterns of neuronal activity, as seen for example in recent views but not the current view of an object. Another situation in which this could be useful is when there is a teacher forcing each neuron to respond, as in a pattern association network, in which two orthogonal input patterns could be learned onto the same output neuron [6,9,72]. We note that in practice, the use of clipping the synaptic weights at a maximum value is especially useful in a heavily loaded network.

To consider this further, there is evidence that some cortical neurons may have a lognormal distribution of synaptic weights [86,87], which implies that neurons have some large weights that may dominate the responses of the neurons. We therefore examined the synaptic weight distribution of neurons in VisNet3 that had learned during the training of invariant object representations. We found that in Layers 2–4 of VisNet3 these trained neurons had synaptic weight distributions that were fitted well by a lognormal distribution. This was found for the usual case with no clipping of synaptic weights, and with clipping using MAX_WEIGHTS = 0.3, in simulations with the parameters as in Fig 2. The difference of the synaptic weight distributions is in the few weights larger than the value of MAX_WEIGHTS, which are limited to 0.3 if MAX_WEIGHTS = 0.3. Without MAX_WEIGHTS being set, the responses of a neuron may be dominated by these relatively few large weights. The implication of having MAX_WEIGHTS set, is that when many objects are represented, the number of synaptic weights used to represent the many different objects can be larger, because a neuron’s response is not dominated by just a few large synaptic weights, which might work for one or a few objects, but not for a large number of objects. In this way, setting the maximum value of synaptic weights may produce some benefits that are not just related to the sparseness of the neuronal representation.

Overall, we propose here that synaptic weight saturation or clipping at a maximum value is an interesting principle of cortical computation that can have advantages in some neuronal networks, and for example in VisNet3 can increase the number of objects that can be learned with invariant representations.

A third advance is in testing VisNet3 with large numbers of training images, to investigate how it scales up, which is important for understanding the biological plausibility of this approach. This helped to show how the numbers of synapses per neuron, and the numbers of neurons in each layer of the network, are related to how many objects can be correctly categorized by the network (Fig 5). Of course, use of the ‘standard competitive net rule’ shown in Equation 5 is also important in increasing the storage capacity of the network, relative to the use of weight normalization. The implication of what is shown in Fig 5 is that if VisNet3 were scaled up to be in the order of a 2 × 2 mm cortical area, that hundreds of objects could be stored with invariant representations in a module of cortex such as this. (The thinking here is based on a number of synapses on each neuron in higher visual cortical regions in the order of 10,000 [6,9,49], and a number of pyramidal cells in a small region of neocortex within 2 mm of approximately 260,000 (if a neuronal density of 30,000/mm^3^ is assumed [6,9]).

The changes to VisNet introduced in VisNet3, make it even more biologically plausible. One of the changes is the new competitive net learning rule implementing heterosynaptic long-term depression that depends on the magnitude of the synaptic weight (Equation 5) to bypass the need for artificial synaptic weight normalization on every neuron to enable equal competition between neurons, and is one improvement. A second improvement is showing that clipping or allowing to saturate synaptic weights at a maximum value, which is highly biological plausible, can actually improve performance particularly in large networks by facilitating more distributed synaptic weight vectors on each neuron.

Although VisNet3 was investigated here for building representations with invariance over views of objects, VisNet can learn in principle any transform of objects, and has been shown to implement translation (shift) invariance, size invariance, lighting invariance, deformation invariance, and even combinations of translation and view invariance [6,9,39,41,45,48,49,65,88–92]. The VisNet architecture is able to do this because it has a short-term memory trace for slow learning that links together the representations across its layers of features and then whole objects, as they transform across a short time period when viewed in the natural world. VisNet3 investigates shape recognition, and therefore does not used color in its training images. However, when color is added to a network such as VisNet, it will greatly improve performance, because colored patches are a further level of description that can be used to distinguish between objects, and could easily be learned by for example having the Gabor filters available in different colors.

VisNet is a generic model of how a feature hierarchy approach combined with a slow learning (trace short-term memory) rule can learn to represent objects, but the ‘objects’ could be of many types, including faces; body parts; objects such as toys, cars, and tools; and even words. Indeed and quite remarkably, a mechanism has been proposed for how letters are combined in what is effectively a feature hierarchy network to gradually build up whole words [93–96]. What does happen is that these types of representation are frequently somewhat segregated into different cortical regions or patches, including in macaques [8,19,21,97–101], and in humans who have a fusiform face area, and just lateral to it a visual word form area, and also patches for tools and body parts [24,38,93,95,96,98,102,103]. One principle here is that topographic maps form in cortical networks with recurrent collateral associatively modifiable connections and lateral inhibition, which have the great advantage of minimizing cortical connection distances and thereby brain size [6,9]. However, even more interestingly than that, other cortical regions are recruited by visual stimuli such as faces, scenes, body parts, and tools, based on their semantics, with for example stationary tools activating visual motion regions such as FST in humans [38]. In each of these systems/patches, networks with generic properties similar to VisNet may be operating, but the effects of brain damage will depend on exactly which patches are damaged with the different types of stimulus material that each processes [6,9].

An advantage of a network like VisNet that has four layers that correspond approximately to V2, V4, posterior inferior temporal cortex, and anterior inferior temporal cortex and that learns with local learning rules is that the neurons in its four layers can be compared with neurons recorded in the brain. Simple properties such as the gradual increase of receptive field size up through the network are evident in VisNet in a similar way to those in the brain. And the tuning of neurons in layer 4 of VisNet to a set of stimuli can be similar to that of neurons in the anterior inferior temporal visual cortex [6,9]. In addition, it has been shown how the receptive fields of neurons in Layer 1 are formed by combinations of the inputs received from the input Gabor filters that correspond to V1 [6,9,41]. But much more could be done in layers 2 and 3 of VisNet to understand how features from earlier layers are combined to help solve object recognition.

Given that VisNet3 is even more biologically plausible given the research described here, we may compare it with approaches that depend on deep learning with non-local synaptic modification rules that are being used as models of how visual object recognition may be implemented in the primate including human brain [61–63]. These models include hierarchical convolutional deep neural networks (HCNN) [61,63], which involve non-biologically plausible operations such as error backpropagation learning, and copying what has been set up in one part of a Layer to all other parts of the same Layer, which is also a non-local operation [9,59,60,104]. The deep learning and convolution neural network approaches are superficially attractive [62], for the responses of neurons in these artificial networks have similarities with the responses of neurons recorded in the primate visual cortical pathways to the inferior temporal visual cortex. But while these similarities are computationally interesting, it is not surprising if artificial neural networks as powerful as deep learning and convolution can be set up to produce these similarities, which do not show that the algorithms used in these artificial network approaches are how the brain implements invariant visual object representation [6].

Key issues for these artificial neural network approaches [62] include the following. One is that the learning rule is not local to the synapses, involving only the presynaptic and the post-synaptic firing rates, but depends on errors artificially computed by for example backpropagation of errors to train the synaptic weights in the hierarchical convolutional deep neural networks (HCNN) [61–63]. Attempts to overcome some of these issues involve for example predictive networks [105], but here too special mechanisms need to be invoked to compute errors locally. Hierarchical convolutional deep neural networks also involve non-biologically plausible operations such as error backpropagation learning, and copying what has been set up in one part of a Layer to all other parts of the same Layer, which is also a non-local operation [9,59,60,62,104].

A second issue it that typically (though not always [106]) a teacher for each output neuron is needed, and nothing like this is evident in the visual cortical regions.

A third issue is lateral propagation of synaptic weights in deep convolution networks [61–63] which is not biologically plausible.

A fourth issue is that with the powerful training of artificial deep networks, the system can become fragile, with alteration of even a few pixels in an image altering the performance of the artificial network, whereas this typically [62] has almost no effect on object recognition in humans, and it would be interesting to test whether VisNet operates more like humans in this respect.

A fifth issue is that the artificial network approaches do not train in a biologically plausible way in that they use just large training sets of images for which categories are typically specified, whereas VisNet capitalizes on the statistics of how images transform in the natural world, and uses these statistics that tend to be produced by the same object over a few seconds as a key part of the training of transform invariant representations of objects, using the trace learning rule [49]. This may be a key point, and may contribute to why these deep HCNNs do not perform in the way that humans do, because the training is so different.

A sixth issue is that VisNet provides utility for the local recurrent collateral synaptic connections within a cortical region that have the potential to support attractor networks [6,9] which are likely to be important in maintaining neuronal activity for periods of a few seconds while an object is being viewed with its different transforms to help implement trace rule learning, whereas local attractor dynamics do not have a key role in most artificial network approaches to visual object recognition [62].

A seventh issue is that the cerebral cortex is able to perform its computations for invariances in networks with just 4 or 5 Layers (see Fig 1). Part of the reason for this is to maximize processing speed, and minimize computation and reaction time [9,64], but it does show that networks with one hundred or more Layers as in some artificial neural networks are not needed to solve the computations involved in transform-invariant object recognition.

An eighth issue is that after an artificial neural network has been trained with error backpropagation and obtains good performance on the training set, if a few more stimuli need to be learned, this can degrade the performance of the network on the already trained images, and there is then a delay until good performance can be achieved again after much more training [107]. This undesirable property does not occur when real human brain networks learn new objects or faces.

A ninth issue is that deep learning models do not fit the psychology of human vision well, partly because the deep learning models typically make predictions about percent success on a database, but do not test particular hypotheses about how the human visual system operates [108]. Many problems arise in the way that deep neural networks process images that are unlike human performance, including that they often classify images based on texture rather than shape; that they classify images based on local rather than global shape; and that they ignore the relations between parts when classifying images [108].

We note that of these 9 issues, VisNet does not have the problems with AI systems in terms of biological plausibility described for issues 1, 2, 3, 5, 6, and probably 7. It will be of interest to see whether in future AI approaches can be developed that seem to be more biologically plausible and therefore to help more directly to elucidate how the brain computes [6,9].

Nor do these artificial network approaches [62] address important issues about how the brain works to solve these computationally demanding tasks, issues such as how synaptic weights are kept bounded in the real brain to be approximately equal on each neuron, nor the computational utility demonstrated here of having the synaptic weights on neurons limited to a biologically plausible maximum value. Other key properties of the VisNet3 model of biologically plausible invariant object recognition described here that may not be incorporated in AI models include the use of sparse connections from layer to layer (which helps each neuron to build different and stable representations); sparse representations (which help with storage capacity); and large numbers of synapses on each neuron which are important in the capacity of the system, as is the large number of neurons in potentially specialized different cortical modules for different types of stimulus such as faces, objects, etc. Another key property of the network described here is that it utilizes only 4 layers or stages of processing after V1 to model the real cortical organization where a few layers or stages of hierarchical processing (Fig 1) ensures that the whole network can respond rapidly. Thus by investigating a biologically plausible network of invariant visual object recognition such as VisNet3, key issues are opened up about how invariant visual object recognition is actually implemented in the primate including human ventral visual system.

What is described here and elsewhere for VisNet [6,9,48,49] may thus it is hoped be useful for developing better artificial neural networks and artificial intelligence. For example, convolutional neural networks are typically trained on very large numbers of single training image exemplars (snapshots) of the classes to be learned, and can fail if a few pixels in the image are altered, implying that they learn pixel-level representations. It is proposed here that training such networks with different transforms of objects would much better enable transform-invariant shape-based representations to be learned, leading to much more powerful performance. Potential limitations of current deep learning methods have also been noted by others [107,109,110], and are developed further [6].

Overall what is described here are key advances in understanding how biologically plausible networks operate to perform massive computational tasks in the cerebral cortex. One key advance is the use of a synaptic modification rule that has long-term potentiation complemented by heterosynaptic long-term depression that depends on the strength of the synapse. This has not been tested previously in a multilayer model of invariant visual object recognition in the ventral visual cortical stream of primates including humans, and removes the need for biologically implausible synaptic weight normalization on each neuron. An implication is that much more experimental research on this type of synaptic modification in the cerebral cortex is indicated. A second advance is that implementing a very biologically plausible upper limit to the strength of synapses can greatly help computationally in at least networks of the type described to improve performance by promoting more distributed sets of synaptic weights on each neuron. This has not been investigated previously before in any model of invariant visual object recognition as far as we know. A third advance is that limiting the contribution of low firing rate neurons to synaptic modification can improve performance. A fourth advance is that we show that with these enhancements, this biologically plausible architecture scales up well for the massive computational task of invariant visual object and face recognition. A fifth contribution is that we raise the issue of the usefulness of biologically plausible approaches to cortical computation such as that described here for invariant visual object and face recognition compared with the usefulness of artificial networks trained by deep learning using error backpropagation perhaps with convolution. The issue arises of how appropriate and useful these artificial neural network approaches are to understanding cortical function, if they use algorithms that are different from those implemented in the brain. This is an extremely timely issue to raise now for discussion, given that it is of course possible to train deep networks with backpropagation of error (rather than a biologically plausible local synaptic learning rule) to emulate some aspects of brain function, but perhaps using completely different algorithms to those used by the brain.

## Supporting information

S1 Appendix Fig A. Convergence in the visual system. Right – as it occurs in the brain. V1, visual cortex area V1; TEO, posterior inferior temporal cortex; TE, anterior inferior temporal cortex (IT). Left– as implemented in VisNet. Convergence through the network is designed to provide fourth layer neurons with information from across the entire input retina. Fig B. Lateral inhibition filter, which was implemented by a Difference of Gaussians filter (see text). Fig C: The filter sampling paradigm. Here each square represents the retinal image presented to the network after being filtered by a Gabor filter of the appropriate orientation sign and frequency. The circles represent the consistent retinotopic coordinates used to provide input to a layer 1 cell. The filters double in spatial frequency towards the reader. Left to right the orientation tuning increases from 0° in steps of 45°, with segregated pairs of positive (P) and negative (N) filter responses.
(PDF)

S1 Code VisNetMat3code. (ZIP)

## References

[1] Perrett DI, Rolls ET, Caan W. Visual neurones responsive to faces in the monkey temporal cortex. Exp Brain Res. 1982;47(3):329–42. doi: 10.1007/BF00239352 7128705

[2] Rolls ET. Neurons in the cortex of the temporal lobe and in the amygdala of the monkey with responses selective for faces. Hum Neurobiol. 1984;3(4):209–22. 6526707

[3] Booth MC, Rolls ET. View-invariant representations of familiar objects by neurons in the inferior temporal visual cortex. Cereb Cortex. 1998;8(6):510–23. doi: 10.1093/cercor/8.6.510 9758214

[4] Perrett DI, Rolls ET, Caan W. Temporal lobe cells of the monkey with visual responses selective for faces. Neurosci Lett. 1979;S3:S358.

[5] Rolls ET. Face neurons. In: Calder AJ, Rhodes G, Johnson MH, Haxby JV, editors. The Oxford Handbook of Face Perception. Oxford: Oxford University Press; 2011. pp. 51–75.

[6] Rolls ET. Brain computations and principles; and AI. Oxford: Oxford University Press; 2026.

[7] Rolls ET. Neuroscience discoveries. Cambridge, MA: MIT Press; 2026.

[8] Hasselmo ME, Rolls ET, Baylis GC, Nalwa V. Object-centered encoding by face-selective neurons in the cortex in the superior temporal sulcus of the monkey. Exp Brain Res. 1989;75(2):417–29. doi: 10.1007/BF00247948 2721619

[9] Rolls ET. Brain Computations and Connectivity. Oxford: Oxford University Press; 2023.

[10] Tovee MJ, Rolls ET, Azzopardi P. Translation invariance in the responses to faces of single neurons in the temporal visual cortical areas of the alert macaque. J Neurophysiol. 1994;72(3):1049–60. doi: 10.1152/jn.1994.72.3.1049 7807195

[11] Rolls ET, Aggelopoulos NC, Zheng F. The receptive fields of inferior temporal cortex neurons in natural scenes. J Neurosci. 2003;23(1):339–48. doi: 10.1523/JNEUROSCI.23-01-00339.2003 12514233 PMC6742126

[12] Rolls ET, Baylis GC. Size and contrast have only small effects on the responses to faces of neurons in the cortex of the superior temporal sulcus of the monkey. Exp Brain Res. 1986;65(1):38–48. doi: 10.1007/BF00243828 3803509

[13] Rolls ET, Baylis GC, Leonard CM. Role of low and high spatial frequencies in the face-selective responses of neurons in the cortex in the superior temporal sulcus in the monkey. Vision Res. 1985;25(8):1021–35. doi: 10.1016/0042-6989(85)90091-4 4071982

[14] Rolls ET. Hippocampal Discoveries: Spatial View Cells, Connectivity, and Computations for Memory and Navigation, in Primates Including Humans. Hippocampus. 2025;35(1):e23666. doi: 10.1002/hipo.23666 39690918 PMC11653063

[15] Rolls ET, Treves A. The neuronal encoding of information in the brain. Prog Neurobiol. 2011;95(3):448–90. doi: 10.1016/j.pneurobio.2011.08.002 21907758

[16] Hesse JK, Tsao DY. The macaque face patch system: a turtle’s underbelly for the brain. Nat Rev Neurosci. 2020;21(12):695–716. doi: 10.1038/s41583-020-00393-w 33144718

[17] Chang L, Tsao DY. The Code for Facial Identity in the Primate Brain. Cell. 2017;169(6):1013–1028.e14. doi: 10.1016/j.cell.2017.05.011 28575666 PMC8088389

[18] Grimaldi P, Saleem KS, Tsao D. Anatomical Connections of the Functionally Defined “Face Patches” in the Macaque Monkey. Neuron. 2016;90(6):1325–42. doi: 10.1016/j.neuron.2016.05.009 27263973 PMC5573145

[19] Tsao D. The Macaque Face Patch System: A Window into Object Representation. Cold Spring Harb Symp Quant Biol. 2014;79:109–14. doi: 10.1101/sqb.2014.79.024950 25943770

[20] Tsao DY, Schweers N, Moeller S, Freiwald WA. Patches of face-selective cortex in the macaque frontal lobe. Nat Neurosci. 2008;11(8):877–9. doi: 10.1038/nn.2158 18622399 PMC8123225

[21] Tsao DY, Freiwald WA, Tootell RBH, Livingstone MS. A cortical region consisting entirely of face-selective cells. Science. 2006;311(5761):670–4. doi: 10.1126/science.1119983 16456083 PMC2678572

[22] Deen B, Koldewyn K, Kanwisher N, Saxe R. Functional Organization of Social Perception and Cognition in the Superior Temporal Sulcus. Cereb Cortex. 2015;25(11):4596–609. doi: 10.1093/cercor/bhv111 26048954 PMC4816802

[23] Liu J, Harris A, Kanwisher N. Perception of face parts and face configurations: an FMRI study. J Cogn Neurosci. 2010;22(1):203–11. doi: 10.1162/jocn.2009.21203 19302006 PMC2888696

[24] Kanwisher N, McDermott J, Chun MM. The fusiform face area: a module in human extrastriate cortex specialized for face perception. J Neurosci. 1997;17(11):4302–11. doi: 10.1523/JNEUROSCI.17-11-04302.1997 9151747 PMC6573547

[25] Freiwald WA. The neural mechanisms of face processing: cells, areas, networks, and models. Curr Opin Neurobiol. 2020;60:184–91. doi: 10.1016/j.conb.2019.12.007 31958622 PMC7017471

[26] Freiwald WA, Tsao DY, Livingstone MS. A face feature space in the macaque temporal lobe. Nat Neurosci. 2009;12(9):1187–96. doi: 10.1038/nn.2363 19668199 PMC2819705

[27] Afraz A, Yamins DLK, DiCarlo JJ. Neural mechanisms underlying visual object recognition. Cold Spring Harb Symp Quant Biol. 2014;79:99–107. doi: 10.1101/sqb.2014.79.024729 26092883

[28] Li N, Dicarlo JJ. Neuronal learning of invariant object representation in the ventral visual stream is not dependent on reward. J Neurosci. 2012;32(19):6611–20. doi: 10.1523/JNEUROSCI.3786-11.2012 22573683 PMC3367428

[29] Rust NC, Dicarlo JJ. Selectivity and tolerance (“invariance”) both increase as visual information propagates from cortical area V4 to IT. J Neurosci. 2010;30(39):12978–95. doi: 10.1523/JNEUROSCI.0179-10.2010 20881116 PMC2975390

[30] DiCarlo JJ, Maunsell JH. Form representation in monkey inferotemporal cortex is virtually unaltered by free viewing. Nat Neurosci. 2000;3(8):814–21. doi: 10.1038/77722 10903575

[31] Desimone R. Face-selective cells in the temporal cortex of monkeys. J Cogn Neurosci. 1991;3(1):1–8. doi: 10.1162/jocn.1991.3.1.1 23964801

[32] Desimone R, Albright TD, Gross CG, Bruce C. Stimulus-selective properties of inferior temporal neurons in the macaque. J Neurosci. 1984;4(8):2051–62. doi: 10.1523/JNEUROSCI.04-08-02051.1984 6470767 PMC6564959

[33] Desimone R, Ungerleider L. Neural mechanisms of visual processing in monkeys. In: Boller F, Grafman J, editors. Handbook of Neuropsychology. New York: Elsevier; 1989. pp. 267–89.

[34] Hoffman KL, Logothetis NK. Cortical mechanisms of sensory learning and object recognition. Philos Trans R Soc Lond B Biol Sci. 2009;364(1515):321–9. doi: 10.1098/rstb.2008.0271 18977728 PMC2674481

[35] Rolls ET, Deco G, Zhang Y, Feng J. Hierarchical organization of the human ventral visual streams revealed with magnetoencephalography. Cereb Cortex. 2023;33(20):10686–701. doi: 10.1093/cercor/bhad318 37689834

[36] Rolls ET, Deco G, Huang C-C, Feng J. Multiple cortical visual streams in humans. Cereb Cortex. 2023;33(7):3319–49. doi: 10.1093/cercor/bhac276 35834308

[37] Rolls ET. Two what, two where, visual cortical streams in humans. Neurosci Biobehav Rev. 2024;160:105650. doi: 10.1016/j.neubiorev.2024.105650 38574782

[38] Rolls ET, Feng J, Zhang R. Selective activations and functional connectivities to the sight of faces, scenes, body parts and tools in visual and non-visual cortical regions leading to the human hippocampus. Brain Struct Funct. 2024;229(6):1471–93. doi: 10.1007/s00429-024-02811-6 38839620 PMC11176242

[39] Rolls ET. Neurophysiological mechanisms underlying face processing within and beyond the temporal cortical visual areas. Philos Trans R Soc Lond B Biol Sci. 1992;335(1273):11–20; discussion 20-1. doi: 10.1098/rstb.1992.0002 1348130

[40] Földiák P. Learning invariance from transformation sequences. Neural Comput. 1991;3(2):194–200. doi: 10.1162/neco.1991.3.2.194 31167302

[41] Wallis G, Rolls ET. Invariant face and object recognition in the visual system. Prog Neurobiol. 1997;51(2):167–94. doi: 10.1016/s0301-0082(96)00054-8 9247963

[42] Deco G, Rolls ET. A neurodynamical cortical model of visual attention and invariant object recognition. Vision Res. 2004;44(6):621–42. doi: 10.1016/j.visres.2003.09.037 14693189

[43] Aggelopoulos NC, Rolls ET. Scene perception: inferior temporal cortex neurons encode the positions of different objects in the scene. Eur J Neurosci. 2005;22(11):2903–16. doi: 10.1111/j.1460-9568.2005.04487.x 16324125

[44] Trappenberg TP, Rolls ET, Stringer SM. Effective Size of Receptive Fields of Inferior Temporal Visual Cortex Neurons in Natural Scenes. In: Dietterich TG, Becker S, Ghahramani Z, editors. Advances in Neural Information Processing Systems 14. Cambridge, MA: The MIT Press; 2002. p. 293–300. doi: 10.7551/mitpress/1120.003.0042

[45] Rolls ET, Webb TJ. Finding and recognizing objects in natural scenes: complementary computations in the dorsal and ventral visual systems. Front Comput Neurosci. 2014;8:85. doi: 10.3389/fncom.2014.00085 25161619 PMC4130325

[46] Rolls ET, Milward T. A model of invariant object recognition in the visual system: learning rules, activation functions, lateral inhibition, and information-based performance measures. Neural Comput. 2000;12(11):2547–72. doi: 10.1162/089976600300014845 11110127

[47] Rolls ET, Stringer SM. Invariant object recognition in the visual system with error correction and temporal difference learning. Network. 2001;12(2):111–29. doi: 10.1088/0954-898x/12/2/302 11405418

[48] Rolls ET. Invariant Visual Object and Face Recognition: Neural and Computational Bases, and a Model, VisNet. Front Comput Neurosci. 2012;6:35. doi: 10.3389/fncom.2012.00035 22723777 PMC3378046

[49] Rolls ET. Learning invariant object and spatial view representations in the brain using slow unsupervised learning. Front Comput Neurosci. 2021;15:686239. doi: 10.3389/fncom.2021.686239 34366818 PMC8335547

[50] Wiskott L, Sejnowski TJ. Slow feature analysis: unsupervised learning of invariances. Neural Comput. 2002;14(4):715–70. doi: 10.1162/089976602317318938 11936959

[51] Franzius M, Sprekeler H, Wiskott L. Slowness and sparseness lead to place, head-direction, and spatial-view cells. PLoS Comput Biol. 2007;3(8):e166. doi: 10.1371/journal.pcbi.0030166 17784780 PMC1963505

[52] Wyss R, König P, Verschure PFMJ. A model of the ventral visual system based on temporal stability and local memory. PLoS Biol. 2006;4(5):e120. doi: 10.1371/journal.pbio.0040120 16605306 PMC1436026

[53] Schönfeld F, Wiskott L. Modeling place field activity with hierarchical slow feature analysis. Front Comput Neurosci. 2015;9:51. doi: 10.3389/fncom.2015.00051 26052279 PMC4441153

[54] Rolls ET, Zhang C, Feng J. Slow semantic learning in the cerebral cortex, and its relation to the hippocampal episodic memory system. Cereb Cortex. 2025;35(5):bhaf107. doi: 10.1093/cercor/bhaf107 40347159

[55] Rumelhart DE, Zipser D. Feature discovery by competitive learning. Cogn Sci. 1985;9:75–112.

[56] Hertz J, Krogh A, Palmer RG. Introduction to the Theory of Neural Computation. Redwood City, CA: Addison-Wesley; 1991.

[57] Oja E. A simplified neuron model as a principal component analyzer. J Math Biol. 1982;15(3):267–73. doi: 10.1007/BF00275687 7153672

[58] Lu W, Du X, Wang J, Zeng L, Ye L, Xiang S, et al. Simulation and assimilation of the digital human brain. Nat Comput Sci. 2024;4(12):890–8. doi: 10.1038/s43588-024-00731-3 39702784

[59] LeCun Y, Bengio Y, Hinton G. Deep learning. Nature. 2015;521(7553):436–44. doi: 10.1038/nature14539 26017442

[60] LeCun Y, Kavukcuoglu K, Farabet C. Convolutional Networks and Applications in Vision. In: 2010 IEEE International Symposium on Circuits and Systems. 2010. pp. 253–6.

[61] Yamins DLK, DiCarlo JJ. Using goal-driven deep learning models to understand sensory cortex. Nat Neurosci. 2016;19(3):356–65. doi: 10.1038/nn.4244 26906502

[62] Kar K, DiCarlo JJ. The quest for an integrated set of neural mechanisms underlying object recognition in primates. Annu Rev Vis Sci. 2024;10(1):91–121. doi: 10.1146/annurev-vision-112823-030616 38950431

[63] Rajalingham R, Issa EB, Bashivan P, Kar K, Schmidt K, DiCarlo JJ. Large-Scale, High-Resolution Comparison of the Core Visual Object Recognition Behavior of Humans, Monkeys, and State-of-the-Art Deep Artificial Neural Networks. J Neurosci. 2018;38(33):7255–69. doi: 10.1523/JNEUROSCI.0388-18.2018 30006365 PMC6096043

[64] Rolls ET. Cerebral Cortex: Principles of Operation. Oxford: Oxford University Press; 2016.

[65] Rolls ET, Mills WPC. Non-accidental properties, metric invariance, and encoding by neurons in a model of ventral stream visual object recognition, VisNet. Neurobiol Learn Mem. 2018;152:20–31. doi: 10.1016/j.nlm.2018.04.017 29723671

[66] Wallis G, Rolls ET, Földiák P. Learning invariant responses to the natural transformations of objects. In: International Joint Conference on Neural Networks. 1993. pp. 1087–90.

[67] Rolls ET, Tovee MJ. Processing speed in the cerebral cortex and the neurophysiology of visual masking. Proc Biol Sci. 1994;257(1348):9–15. doi: 10.1098/rspb.1994.0087 8090795

[68] Rolls ET. Consciousness absent and present: a neurophysiological exploration. Prog Brain Res. 2004;144:95–106. doi: 10.1016/S0079-6123(03)14406-8 14650842

[69] Gerstner W, Kistler WM, Naud R, Paninski L. Neuronal dynamics: From single neurons to networks and models of cognition. Cambridge: Cambridge University Press; 2014.

[70] Treves A, Rolls ET. What determines the capacity of autoassociative memories in the brain? Network: Comput Neural Syst. 1991;2(4):371–97. doi: 10.1088/0954-898x_2_4_004

[71] Treves A. Dilution and sparse coding in threshold-linear nets. J Phys A: Math Gen. 1991;24(1):327–35. doi: 10.1088/0305-4470/24/1/038

[72] Rolls ET, Treves A. The relative advantages of sparse versus distributed encoding for associative neuronal networks in the brain. Network: Comput Neural Syst. 1990;1(4):407–21. doi: 10.1088/0954-898x_1_4_002

[73] Rolls ET, Tovee MJ. Sparseness of the neuronal representation of stimuli in the primate temporal visual cortex. J Neurophysiol. 1995;73(2):713–26. doi: 10.1152/jn.1995.73.2.713 7760130

[74] Franco L, Rolls ET, Aggelopoulos NC, Jerez JM. Neuronal selectivity, population sparseness, and ergodicity in the inferior temporal visual cortex. Biol Cybern. 2007;96(6):547–60. doi: 10.1007/s00422-007-0149-1 17410377

[75] Hagena H, Manahan-Vaughan D. Interplay of hippocampal long-term potentiation and long-term depression in enabling memory representations. Philos Trans R Soc Lond B Biol Sci. 2024;379(1906):20230229. doi: 10.1098/rstb.2023.0229 38853558 PMC11343234

[76] Willshaw DJ, von der Malsburg C. How patterned neural connections can be set up by self-organization. Proc R Soc Lond B Biol Sci. 1976;194(1117):431–45. doi: 10.1098/rspb.1976.0087 12510

[77] Grossberg S. Adaptive pattern classification and universal recoding: II. Feedback, expectation, olfaction, illusions. Biol Cybern. 1976;23(4):187–202. doi: 10.1007/BF00340335 963125

[78] Miller KD, MacKay DJC. The role of constraints in Hebbian learning. Neural Computation. 1994;6(1):100–26. doi: 10.1162/neco.1994.6.1.100

[79] Daugman JG. Complete discrete 2-D Gabor transforms by neural networks for image analysis and compression. IEEE Trans Acoust, Speech, Signal Processing. 1988;36(7):1169–79. doi: 10.1109/29.1644

[80] Rolls ET, Turova TS. Visual cortical networks for “What” and “Where” to the human hippocampus revealed with dynamical graphs. Cereb Cortex. 2025;35(5):bhaf106. doi: 10.1093/cercor/bhaf106 40347158

[81] Geusebroek JM, Burghouts GJ, Smeulders AWM. The Amsterdam Library of Object Images. Int J Comput Vis. 2005;61(1):103–12.

[82] Wang XJ. Synaptic basis of cortical persistent activity: the importance of NMDA receptors to working memory. J Neurosci. 1999;19(21):9587–603. doi: 10.1523/JNEUROSCI.19-21-09587.1999 10531461 PMC6782911

[83] Bienenstock EL, Cooper LN, Munro PW. Theory for the development of neuron selectivity: orientation specificity and binocular interaction in visual cortex. J Neurosci. 1982;2(1):32–48. doi: 10.1523/JNEUROSCI.02-01-00032.1982 7054394 PMC6564292

[84] Rolls ET, Treves A. A theory of hippocampal function: New developments. Prog Neurobiol. 2024;238:102636. doi: 10.1016/j.pneurobio.2024.102636 38834132

[85] Rolls ET. Hippocampal Revolutions. Neurosci Biobehav Rev. 2026;180:106492. doi: 10.1016/j.neubiorev.2025.106492 41308965

[86] Melander JB, Nayebi A, Jongbloets BC, Fortin DA, Qin M, Ganguli S, et al. Distinct in vivo dynamics of excitatory synapses onto cortical pyramidal neurons and parvalbumin-positive interneurons. Cell Rep. 2021;37(6):109972. doi: 10.1016/j.celrep.2021.109972 34758304 PMC8631347

[87] Loewenstein Y, Kuras A, Rumpel S. Multiplicative dynamics underlie the emergence of the log-normal distribution of spine sizes in the neocortex in vivo. J Neurosci. 2011;31(26):9481–8. doi: 10.1523/JNEUROSCI.6130-10.2011 21715613 PMC6623170

[88] Webb TJ, Rolls ET. Deformation-specific and deformation-invariant visual object recognition: pose vs. identity recognition of people and deforming objects. Front Comput Neurosci. 2014;8:37. doi: 10.3389/fncom.2014.00037 24744725 PMC3978248

[89] Rolls ET, Stringer SM. Invariant visual object recognition: a model, with lighting invariance. J Physiol Paris. 2006;100(1–3):43–62. doi: 10.1016/j.jphysparis.2006.09.004 17071062

[90] Perry G, Rolls ET, Stringer SM. Spatial vs temporal continuity in view invariant visual object recognition learning. Vision Res. 2006;46(23):3994–4006. doi: 10.1016/j.visres.2006.07.025 16996556

[91] Stringer SM, Rolls ET. Invariant object recognition in the visual system with novel views of 3D objects. Neural Comput. 2002;14(11):2585–96. doi: 10.1162/089976602760407982 12433291

[92] Elliffe MCM, Rolls ET, Stringer SM. Invariant recognition of feature combinations in the visual system. Biol Cybern. 2002;86(1):59–71. doi: 10.1007/s004220100284 11924570

[93] Agrawal A, Dehaene S. Cracking the neural code for word recognition in convolutional neural networks. PLoS Comput Biol. 2024;20(9):e1012430. doi: 10.1371/journal.pcbi.1012430 39241019 PMC11410253

[94] Rajalingham R, Kar K, Sanghavi S, Dehaene S, DiCarlo JJ. The inferior temporal cortex is a potential cortical precursor of orthographic processing in untrained monkeys. Nat Commun. 2020;11(1):3886. doi: 10.1038/s41467-020-17714-3 32753603 PMC7403350

[95] Vinckier F, Dehaene S, Jobert A, Dubus JP, Sigman M, Cohen L. Hierarchical coding of letter strings in the ventral stream: dissecting the inner organization of the visual word-form system. Neuron. 2007;55(1):143–56. doi: 10.1016/j.neuron.2007.05.031 17610823

[96] Dehaene S, Cohen L, Sigman M, Vinckier F. The neural code for written words: a proposal. Trends Cogn Sci. 2005;9(7):335–41. doi: 10.1016/j.tics.2005.05.004 15951224

[97] Baylis GC, Rolls ET, Leonard CM. Functional subdivisions of the temporal lobe neocortex. J Neurosci. 1987;7(2):330–42. doi: 10.1523/JNEUROSCI.07-02-00330.1987 3819816 PMC6568924

[98] Tsao DY, Moeller S, Freiwald WA. Comparing face patch systems in macaques and humans. Proc Natl Acad Sci U S A. 2008;105(49):19514–9. doi: 10.1073/pnas.0809662105 19033466 PMC2614792

[99] Harries MH, Perrett DI. Visual processing of faces in temporal cortex: physiological evidence for a modular organization and possible anatomical correlates. J Cogn Neurosci. 1991;3(1):9–24. doi: 10.1162/jocn.1991.3.1.9 23964802

[100] Perrett DI, Smith PA, Mistlin AJ, Chitty AJ, Head AS, Potter DD, et al. Visual analysis of body movements by neurones in the temporal cortex of the macaque monkey: a preliminary report. Behav Brain Res. 1985;16(2–3):153–70. doi: 10.1016/0166-4328(85)90089-0 4041214

[101] Hasselmo ME, Rolls ET, Baylis GC. The role of expression and identity in the face-selective responses of neurons in the temporal visual cortex of the monkey. Behav Brain Res. 1989;32(3):203–18. doi: 10.1016/s0166-4328(89)80054-3 2713076

[102] Spiridon M, Fischl B, Kanwisher N. Location and spatial profile of category-specific regions in human extrastriate cortex. Hum Brain Mapp. 2006;27(1):77–89. doi: 10.1002/hbm.20169 15966002 PMC3264054

[103] Spiridon M, Kanwisher N. How distributed is visual category information in human occipito-temporal cortex? An fMRI study. Neuron. 2002;35(6):1157–65. doi: 10.1016/s0896-6273(02)00877-2 12354404

[104] Bengio Y, Goodfellow I, Courville A. Deep learning. Massachusetts, USA: MIT Press; 2017.

[105] Song Y, Lukasiewicz T, Xu Z, Bogacz R. Can the Brain Do Backpropagation? -Exact Implementation of Backpropagation in Predictive Coding Networks. Adv Neural Inf Process Syst. 2020;33:22566–79. 33840988 PMC7610561

[106] Zhuang C, Yan S, Nayebi A, Schrimpf M, Frank MC, DiCarlo JJ, et al. Unsupervised neural network models of the ventral visual stream. Proc Natl Acad Sci U S A. 2021;118(3):e2014196118. doi: 10.1073/pnas.2014196118 33431673 PMC7826371

[107] Song Y, Millidge B, Salvatori T, Lukasiewicz T, Xu Z, Bogacz R. Inferring neural activity before plasticity as a foundation for learning beyond backpropagation. Nat Neurosci. 2024;27(2):348–58. doi: 10.1038/s41593-023-01514-1 38172438 PMC7615830

[108] Bowers JS, Malhotra G, Dujmović M, Llera Montero M, Tsvetkov C, Biscione V, et al. Deep problems with neural network models of human vision. Behav Brain Sci. 2022;46:e385. doi: 10.1017/S0140525X22002813 36453586

[109] Plebe A, Grasso G. The unbearable shallow understanding of deep learning. Minds Mach. 2019;29(4):515–53. doi: 10.1007/s11023-019-09512-8

[110] Sejnowski TJ. The unreasonable effectiveness of deep learning in artificial intelligence. Proc Natl Acad Sci U S A. 2020;117(48):30033–8. doi: 10.1073/pnas.1907373117 31992643 PMC7720171

