# Supplementary material for: Invariant visual object and face learning in the ventral cortical visual pathway: A biologically plausible model
Source: PLoS Comput Biol. 2026 Feb 11;22(2):e1013959. doi: 10.1371/journal.pcbi.1013959 (PMC12916060; doi:10.1371/journal.pcbi.1013959)
Supplement: S1 Appendix — Fig A. Convergence in the visual system. Right – as it occurs in the brain. V1, visual cortex area V1; TEO, posterior inferior temporal cortex; TE, anterior inferior temporal cortex (IT). Left– as implemented in VisNet. Convergence through the network is designed to provide fourth layer neurons with information from across the entire input retina. Fig B. Lateral inhibition filter, which was implemented by a Difference of Gaussians filter (see text). Fig C: The filter sampling paradigm. Here each square represents the retinal image presented to the network after being filtered by a Gabor filter of the appropriate orientation sign and frequency. The circles represent the consistent retinotopic coordinates used to provide input to a layer 1 cell. The filters double in spatial frequency towards the reader. Left to right the orientation tuning increases from 0° in steps of 45°, with segregated pairs of positive (P) and negative (N) filter responses. (PDF) [file pcbi.1013959.s001.pdf]

Invariant visual object and face learning in the ventral cortical visual pathway: a biologically plausible model

Supplementary Material

PLoS Computational Biology (2026)
doi: 10.1371/journal.pcbi.1013959

Chenfei Zhang (1), Edmund T Rolls (1,2,3,*) and Jianfeng Feng (1,3)
1 Institute for the Science and Technology of Brain-Inspired Intelligence, Fudan University, Shanghai, China
2 Oxford Centre for Computational Neuroscience, Oxford, United Kingdom
3 University of Warwick, Department of Computer Science, Coventry, United Kingdom
* https:\\www.oxcns.org Edmund.Rolls@oxcns.org

This Supplementary Material provides a description of the architecture of VisNet3. This document also contains Supplementary Tables A, B and C which provide the parameters used in some of the simulations described in the paper [1]. This document also contains a guide to the Matlab code for VisNet3, which is made available in association with this paper [1].

Descriptions of previous research with VisNet and its architecture, and of the neurophysiology of the inferior temporal visual cortex which VisNet3 models, are available [2, 3, 4, 5].

1 The architecture of VisNet

Fundamental elements of Rolls' theory for how cortical networks might implement invariant visual object recognition are described in detail elsewhere [6, 2, 3, 4, 5], provide the basis for the design of VisNet3, and can be summarized as:

- A series of competitive networks, organized in hierarchical layers, exhibiting mutual inhibition over a short range within each layer. These networks allow combinations of features or inputs occurring in a given spatial arrangement to be learned by neurons, ensuring that higher-order spatial properties of the input stimuli are represented in the network.
- A convergent series of connections from a localized population of cells in preceding layers to each cell of the following layer, thus allowing the receptive field size of cells to increase through the visual processing areas or layers.
- A modified Hebb-like learning rule incorporating a temporal trace of each cell's previous activity that enables the neurons to learn transform invariances.

The first two elements of Rolls' theory [5] are used to constrain the general architecture of a network model, VisNet, of the processes just described that is intended to learn invariant representations of objects. The simulation results described in this paper using VisNet show that invariant representations can be learned by the architecture. It is moreover shown that successful learning depends crucially on the use of the modified Hebb rule. The general architecture simulated in VisNet, and the way in which it allows natural images to be used as stimuli, has been chosen to enable some comparisons of neuronal responses in the network and in the brain to similar stimuli to be made.

1.1 The short term memory trace synaptic learning rule

The learning rule implemented in the VisNet simulations utilizes the spatio-temporal constraints placed upon the behaviour of 'real-world' objects to learn about natural object transformations. The concept is that primates typically look at an object for 1 to a few seconds, and in that time the natural statistics of the world will provide several transforms of that object, which can be used to learn the different transforms of that object to produce a transform-invariant neural representation. By presenting consistent sequences of transforming objects the neurons in the network can learn to respond to the same object through all of its naturally transformed states, as described in early research [7, 6, 8, 2], with many further developments [9, 10, 3, 4, 5]. The learning rule incorporates a decaying trace of previous cell activity and is henceforth referred to simply as the 'trace' learning rule. The learning paradigm we describe here is intended in principle to enable learning of any of the transforms tolerated by inferior temporal cortex neurons, including position, size, view, lighting, and spatial frequency [6, 11, 12, 5, 3, 4, 5].

To clarify the reasoning behind this point, consider the situation in which a single neuron is strongly activated by a stimulus forming part of a real world object. The trace of this neuron's activation will then gradually decay over a time period in the order of 0.5 s. If, during this limited time window, the net is presented with a transformed version of the original stimulus then not only will the initially active afferent synapses modify onto the neuron, but so also will the synapses activated by the transformed version of this stimulus. In this way the neuron will learn to respond

to either appearance of the original stimulus. Making such associations works in practice because it is very likely that within short time periods different aspects of the same object will be being viewed. The neuron will not, however, tend to make spurious links across stimuli that are part of different objects because of the unlikelihood in the real world of one object consistently following another.

Various biological bases for this temporal trace have been advanced as follows:

- The persistent firing of neurons for as long as 100–400 ms observed after presentations of stimuli for 16 ms [13] could provide a time window within which to associate subsequent images. Maintained activity may potentially be implemented by recurrent connections between as well as within cortical areas [14, 12, 5]. [The prolonged firing of inferior temporal cortex neurons during memory delay periods of several seconds, and associative links reported to develop between stimuli presented several seconds apart [15, 16] are on too long a time scale to be immediately relevant to the present theory. In fact, associations between visual events occurring several seconds apart would, under *normal* environmental conditions, be detrimental to the operation of a network of the type described here, because they would probably arise from different objects. In contrast, the system described benefits from associations between visual events that occur close in time (typically within 1 s), as they are likely to be from the same object given the statistics of the inputs being received from the natural world.]

- The binding period of glutamate in the NMDA channels, which may last for 100 ms or more, may implement a trace rule by producing a narrow time window over which the *average* activity at each presynaptic site affects learning [6, 17, 18, 19, 20].

- Strengthening (long-term potentiation, LTP) or weakening (long-term depression, LTD) of glutamatergic synapses depends on the post-synaptic influx of calcium (Ca^{2+}): weak influx leads to LTD, while strong, transient influx causes LTP. The voltage-dependent NMDA receptors are the main source of Ca^{2+} influx, but they will only open if a post-synaptic depolarisation coincides with pre-synaptic neurotransmitter release. The interplay between the pre-synaptic neurotransmitter release and the post-synaptic membrane potential leads to distinct Ca^{2+} time-courses, which in turn lead to the change in synaptic strength where the timecourse can be 100 ms or more [21].

The trace update rule used in the baseline simulations of VisNet [2] is equivalent to both Földiák's used in the context of translation invariance [8] and to an earlier rule [22] explored in the context of modelling the temporal properties of classical conditioning, and can be summarized as follows:

$$\delta w_j = \alpha \overline{y}^{\tau} x_j \qquad (1)$$

where

$$\overline{y}^{\tau} = (1-\eta) y^{\tau} + \eta \overline{y}^{\tau-1} \qquad (2)$$

and

x_j:	j^{th} input to the neuron.	y:	Output from the neuron.
$\overline{y}^{\tau}$:	Trace value of the output of the neuron at time step τ.	α:	Learning rate. Annealed between unity and zero.
w_j:	Synaptic weight between j^{th} input and the neuron.	η:	Trace value. The optimal value varies with presentation sequence length.

1.2 Synaptic weight normalisation or scaling in VisNet3

In a competitive network, it is important that all neurons compete on an equal basis, so that different neurons learn to respond to different inputs, and similar inputs are allocated to the same

neuron, so that categorisation is performed usefully [5]. The usual way in which this is implemented in a competitive net is that after learning with a Hebbian associative rule or one with a short term memory trace in the post-synaptic term y such as that in Equation 1, the length of the vector of synaptic weights on a neuron is set to one [23, 24, 25, 5]. Given that a Hebbian rule will always increase synaptic weights if the presynaptic and postsynaptic firing rates are greater than 0, the synaptic modification will increase some synaptic weights. The weight normalisation (setting the sum of the squares of the weights = 1) will then decrease the weights usefully [25, 5]. That is what is implemented in previous implementations of VisNet, by dividing the synaptic weight vector on a neuron by the length of its synaptic weight vector after its synapses have received an update [3, 4, 5].

However, setting the length of the synaptic weight vector on each neuron is not very biologically plausible. So for VisNet3 [1] we introduce an alternative method of allowing each neuron to compete on an equal basis by using a learning rule that allows synaptic weights to decrease in value if they are on a strongly activated neuron, and the current weight is larger than the presynaptic term. This provides for heterosynaptic long-term depression, which as many experimentalists will know, is easier to obtain if the synaptic weights are already high, in addition to long-term potentiation. The rule we introduce for VisNet3 is

$$\delta w_j = \alpha y(x_j - w_j) \quad (3)$$

This rule was used in different applications previously [26, 27, 25], and has been termed the 'standard competitive net learning rule' [25]. In the main text of this paper [1] we compare the operation of VisNet3 using this 'standard competitive network rule' shown in Equation 3, with the weight normalisation used in VisNet [3, 4], and with the Oja rule [28, 25] shown in Equation 4

$$\delta w_j = \alpha y(x_j - y w_j) \quad (4)$$

which though somewhat similar to what is shown in Equation 3 can normalise the synaptic weight vector and is we suggest less biologically plausible than the Rule shown in Equation 3. It is shown in the main text [1] that training VisNet3 with Equation 3 produces somewhat better performance than with Equation 4, and much better than that achieved with the associative learning and weight normalisation used in VisNet.

1.3 Limiting the maximum synaptic weight on a neuron

With normal Hebbian learning using a rule like that shown in Equation 1 (but without a short-term memory trace on the post-synaptic term y) some synaptic weights might continue to increase to high values, especially if some features are present in different objects. It seems biologically implausible that synaptic weights could grow without bound, so we have investigated limiting the maximum value that a synaptic weight on a neuron can reach (set with $MAX - WEIGHT$ in the Matlab code for VisNet3). We in fact propose that this could be beneficial, by encouraging neurons not to rely on a few strong synaptic weights from high-firing inputs to the neuron, but to grow weights from a number of inputs, in order to increase the sampling of information from the preceding layer by producing a more distributed representation for what is learned by each neuron. We show in the simulations presented in the text of the paper [1] that this can be useful for increasing the memory capacity in at least large network versions of VisNet3. This process is typically combined with other methods to scale the weights on a neuron, such as the procedure implemented in Equation 3.

1.4 The network implemented in VisNet3

The VisNet3 network is designed as a series of hierarchical, convergent, competitive networks, in accordance with the hypotheses advanced above. The actual network consists of a series of four layers, constructed such that the convergence of information from the network's input layer can potentially influence firing in any single neuron in the final layer – see Fig. A. This is consistent

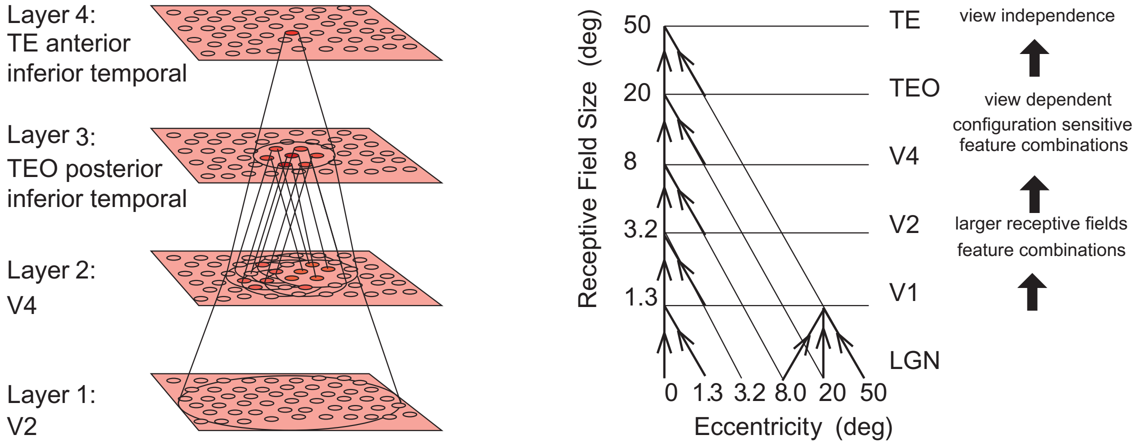

Figure A: Convergence in the visual system. Right – as it occurs in the brain. V1, visual cortex area V1; TEO, posterior inferior temporal cortex; TE, anterior inferior temporal cortex (IT). Left – as implemented in VisNet. Convergence through the network is designed to provide fourth layer neurons with information from across the entire input retina.

with what is found in the primate visual system [29, 6, 5]. Layer 1 of VisNet3 corresponds to V2, and receives inputs from V1. Layer 2 of VisNet3 corresponds to V4, Layer 3 to the posterior inferior temporal visual cortex region TEO in macaques, and Layer 4 of VisNet3 corresponds to the anterior temporal lobe visual cortex region TE in macaques (Fig. A). The forward connections to a cell in one layer are derived from a topologically related and confined region of the preceding layer. The choice of whether a connection between neurons in adjacent layers exists or not is based upon a Gaussian distribution of connection probabilities which roll off radially from the focal point of connections for each neuron. (A minor extra constraint precludes the repeated connection of any pair of cells.) In particular, the forward connections to a cell in one layer come from a small region of the preceding layer defined by the radius in Table A which will contain approximately 67% of the connections from the preceding layer. The radii are scaled up linearly for larger versions of VisNet3. Table A shows the dimensions and default parameters for a small version of VisNet3. Table B shows the dimensions and default parameters for a large version of VisNet, with 256×256 neurons in each layer, and up to 2000 synapses per neuron.

Table A: VisNet3 dimensions and parameters[1]

	Dimensions	# Connections	Radius
Layer 4	32x32	200	7
Layer 3	32x32	200	7
Layer 2	32x32	200	7
Layer 1	32x32	340	15
Input layer	256x256x32	–	–

Figure A shows the general convergent network architecture used. Localization and limitation of connectivity in the network is intended to mimic cortical connectivity, partially because of the clear retention of retinal topology through early visual cortical regions. This architecture also encourages the gradual combination of features from layer to layer which has relevance to the binding problem [3, 4, 5].

To avoid issues at the edges of VisNet, the connectivity wraps into a toroid, such that connections map back onto the network from opposite sides and from the top and bottom. This wrapping happens at all four layers of the network, and in the way an image on the 'retina' is mapped to the input filters. This solution has the advantage of making all of the boundaries effectively invisible to the network, as described previously [2].

[1]Notes for Table A on the default parameters. Where there are 4 values, these are for Layers 1-4 respectively.
Learning Rule: Standard Competitive Net, Equation 3.
Learning Rate $\alpha = =$ [0.025 0.025 0.025 0.025].
SPARSENESS = [0.01 0.01 0.01 0.01]
ETA = [0.0 0.8 0.8 0.8] the trace rule value for each layer.
TrainEpochs = [20 20 20 20] the number of training epochs.
MAX-WEIGHTS if clipped: [0.1 0.1 0.1 NotClipped]
BETA = [10 10 10 10] for the sigmoid activation function.
Number of training objects: 20, each with 9 views.

Table B: VisNet3 dimensions and parameters for a larger version[2]

	Dimensions	# Connections	Square
Layer 4	256x265	1000	11*8*2+1
Layer 3	256x256	1000	11*8*2+1
Layer 2	256x256	1000	11*8*2+1
Layer 1	256x256	340	15*2+1
Input layer	256x256x32	–	–

1.5 Competition and lateral inhibition

In order to act as a competitive network some form of mutual inhibition is required within each layer, which should help to ensure that all stimuli presented are evenly represented by the neurons in each layer [5]. This is implemented in VisNet by a form of lateral inhibition. The idea behind the lateral inhibition, apart from this being a property of cortical architecture in the brain, is to prevent too many neurons that receive inputs from a similar part of the preceding layer responding to the same activity patterns. One purpose of the lateral inhibition is to ensure that different receiving neurons code for different inputs. This is important in reducing redundancy [5]. The lateral inhibition is conceived as operating within a radius that is similar to that of the region within which a neuron receives converging inputs from the preceding layer (because activity in one zone of topologically organized processing within a layer should not inhibit processing in another zone in the same layer, concerned perhaps with another part of the image) [2, 9]. The lateral inhibition is implemented in VisNet3 by convolving the firing rates in a layer with a 2D difference of Gaussian filter with the general form illustrated in Fig. B. The parameters for 32x32 VisNet3 are a Gaussian with width 0.2 from which is subtracted a Gaussian with width 4. This provides a filter that does not alter the mean firing rate. The size of the filter may be scaled up for sizes of VisNet3 larger than 32x32.

A sigmoid activation function is used in VisNet3, with the threshold or bias α used to set the sparseness of the firing rate representation in a layer to implement a form of competition between the neurons. The sigmoid was calculated as

$$y = \mathrm{f}^{\mathrm{sigmoid}}(r) = \frac{1}{1 + e^{-2\beta(r-\alpha)}} \tag{5}$$

where r is the activation (or firing rate) of the neuron after the lateral inhibition, y is the firing rate after the contrast enhancement produced by the activation function, and β is the slope or gain and α is the threshold or bias of the activation function. The sigmoid bounds the firing rate between 0 and 1 so global scaling is not required.

The (population) sparseness a of the firing within a layer that was used to set α is defined [14, 30, 31, 5] as:

$$a = \frac{(\sum_i y_i/n)^2}{\sum_i y_i^2/n} \tag{6}$$

where n is the number of neurons in the layer. To set the sparseness to a given value, e.g. 0.01, the threshold or bias of the activation function α is set to the sparseness required. The sparseness

[2]Notes for Table B on the default parameters. Where there are 4 values, these are for Layers 1-4 respectively. The column headed 'Square' indicates that for the large scale simulations, the inputs came from a square region of the preceding layer, not the usual 2D Gaussian region.
Learning Rule: Standard Competitive Net, Equation 3.
Learning Rate α = [0.005 0.005 0.005 0.005].
SPARSENESS = [0.0025 0.0025 0.0025 0.0025]
ETA = [0.0 0.8 0.8 0.8] the trace rule value for each layer.
TrainEpochs = [50 50 50 50] the number of training epochs.
MAX-WEIGHTS [0.06 0.06 0.06 Not set]
BETA = [100 100 100 100] for the sigmoid activation function.
Number of training objects: 50 - 800, each with 9 views.

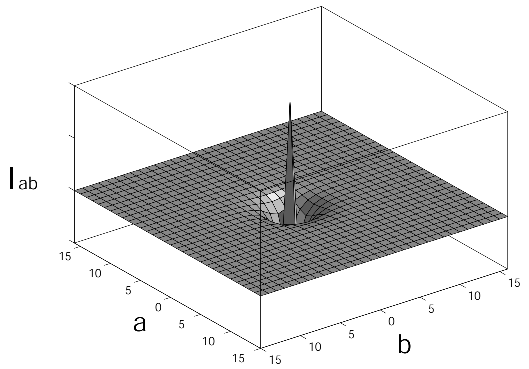

Figure B: Lateral inhibition filter, which was implemented by a Difference of Gaussians filter (see text).

value used in the simulations was 0.01 unless otherwise stated. The value used for β was 10.

1.6 The input to VisNet

VisNet is provided with a set of input filters that can be applied to an image to produce inputs to the network which correspond to those provided by simple cells in visual cortical area 1 (V1) (Fig. C). The purpose of this is to enable within VisNet the more complicated response properties of cells between V1 and the inferior temporal cortex (IT) to be investigated, using as inputs natural stimuli such as those that could be applied to the retina of the real visual system. This is to facilitate comparisons between the activity of neurons in VisNet and those in the real visual system, to the same stimuli. In VisNet no attempt is made to train the response properties of simple cells, but instead we start with a defined series of filters to perform fixed feature extraction to a level equivalent to that of simple cells in V1, as have other researchers [32, 33, 34], because we wish to simulate the more complicated response properties of cells between V1 and the inferior temporal cortex (IT).

Gabor filters produce good results with VisNet [35], and are what is implemented in VisNet3. Following [36] the receptive fields of the simple cell-like input neurons are modelled by 2D-Gabor functions. The Gabor receptive fields have five degrees of freedom given essentially by the product of an elliptical Gaussian and a complex plane wave. The first two degrees of freedom are the 2D-locations of the receptive field's centre; the third is the size of the receptive field; the fourth is the orientation of the boundaries separating excitatory and inhibitory regions; and the fifth is the symmetry. This fifth degree of freedom is given in the standard Gabor transform by the real and imaginary part, i.e by the phase of the complex function representing it, whereas in a biological context this can be done by combining pairs of neurons with even and odd receptive fields. This design is supported by experimental work [37], which found simple cells in quadrature-phase pairs. Even more, Daugman [36] proposed that an ensemble of simple cells is best modelled as a family of 2D-Gabor wavelets sampling the frequency domain in a log-polar manner as a function of eccentricity. Experimental neurophysiological evidence constrains the relation between the free parameters that define a 2D-Gabor receptive field [38]. There are three constraints fixing the relation between the width, height, orientation, and spatial frequency [39]. The first constraint posits that the aspect ratio of the elliptical Gaussian envelope is 2:1. The second constraint postulates that the plane wave tends to have its propagating direction along the short axis of the elliptical Gaussian. The third constraint assumes that the half-amplitude bandwidth of the frequency response is about 1 to 1.5 octaves along the optimal orientation. Further, we assume that the mean is zero in order to have an admissible wavelet basis [39].

In more detail, the Gabor filters are constructed as follows [35]. We consider a pixelized grey-scale image given by a $N \times N$ matrix $\Gamma^{\text{orig}}_{ij}$. The subindices ij denote the spatial position of the

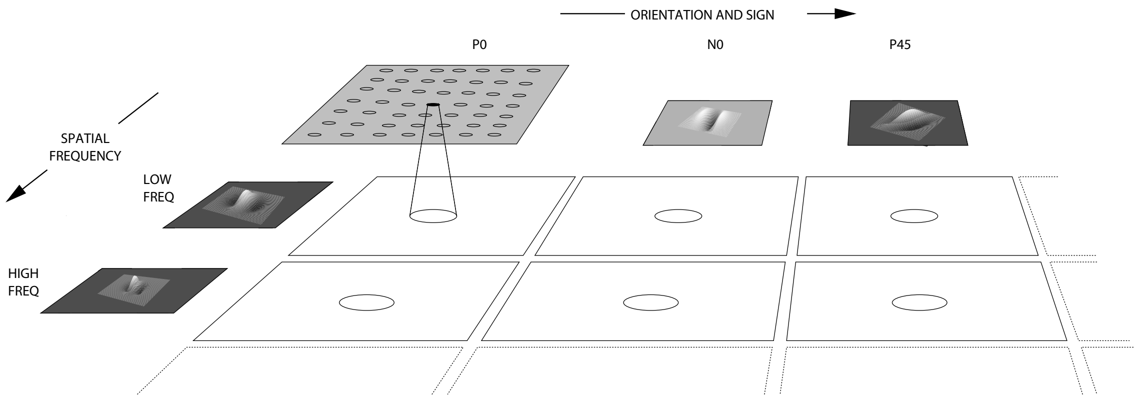

Figure C: The filter sampling paradigm. Here each square represents the retinal image presented to the network after being filtered by a Gabor filter of the appropriate orientation sign and frequency. The circles represent the consistent retinotopic coordinates used to provide input to a layer 1 cell. The filters double in spatial frequency towards the reader. Left to right the orientation tuning increases from 0° in steps of 45°, with segregated pairs of positive (P) and negative (N) filter responses.

pixel. Each pixel value is given a grey level brightness value coded in a scale between 0 (black) and 255 (white). The first step in the preprocessing consists of removing the DC component of the image (i.e. the mean value of the grey-scale intensity of the pixels). (The equivalent in the brain is the low-pass filtering performed by the retinal ganglion cells and lateral geniculate cells. The visual representation in the LGN is essentially a contrast invariant pixel representation of the image, i.e. each neuron encodes the relative brightness value at one location in visual space referred to the mean value of the image brightness.) We denote this contrast-invariant LGN representation by the $N \times N$ matrix Γ_{ij} defined by the equation

$$\Gamma_{ij} = \Gamma_{ij}^{\text{orig}} - \frac{1}{N^2} \sum_{i=1}^{N} \sum_{j=1}^{N} \Gamma_{ij}^{\text{orig}}. \tag{7}$$

Feedforward connections to a layer of V1 neurons perform the extraction of simple features like bars at different locations, orientations and sizes. Realistic receptive fields for V1 neurons that extract these simple features can be represented by 2D-Gabor wavelets. Lee [39] derived a family of discretized 2D-Gabor wavelets that satisfy the wavelet theory and the neurophysiological constraints for simple cells mentioned above. They are given by an expression of the form

$$G_{pqkl}(x, y) = a^{-k} \Psi_{\Theta_l}(a^{-k}(x - 2p), a^{-k}(y - 2q)) \tag{8}$$

where

$$\Psi_{\Theta_l} = \Psi(x \cos(l\Theta_0) + y \sin(l\Theta_0), -x \sin(l\Theta_0) + y \cos(l\Theta_0)), \tag{9}$$

and the mother wavelet is given by

$$\Psi(x, y) = \frac{1}{\sqrt{2\pi}} e^{-\frac{1}{8}(4x^2+y^2)} [e^{i\kappa x} - e^{-\frac{\kappa^2}{2}}]. \tag{10}$$

In the above equations $\Theta_0 = \pi/L$ denotes the step size of each angular rotation; l the index of rotation corresponding to the preferred orientation $\Theta_l = l\pi/L$; k denotes the octave; and the indices pq the position of the receptive field centre at $c_x = p$ and $c_y = q$. In this form, the receptive fields at all levels cover the spatial domain in the same way, i.e. by always overlapping the receptive fields in the same fashion. In the model we use $a = 2$, $b = 1$ and $\kappa = \pi$ corresponding

to a spatial frequency bandwidth of one octave. It is possible in VisNet to use both symmetric and asymmetric filters (as both are present in V1 [40]); with the angular spacing between the different orientations set to 45 degrees; and with 8 filter frequencies spaced one octave apart starting with 0.5 cycles per pixel, and with the sampling from the spatial frequencies set as shown in Table C.

Cells of Layer 1 receive a topologically consistent, localized, random selection of the filter responses in the input layer, under the constraint that each cell samples every filter spatial frequency and receives a constant number of inputs. Figure C shows pictorially the general filter sampling paradigm.

Table C: VisNet layer 1 connectivity. The frequency is in cycles per pixel.

Frequency	0.5	0.25	0.125	0.0625
# Connections	256	64	16	4

Of the 340 connections to each neuron in Layer 1, the number to each frequency group in VisNet3 is as shown in Table C. In VisNet3 by default only even symmetric – 'bar detecting' – filter shapes are used, which take the form of Gabor filters that simulate V1, the primary visual cortex.

1.7 Measures for network performance

Measures of network performance based on information theory and similar to those used in the analysis of the firing of real neurons in the brain [5, 31] are described for VisNet [9, 3, 5] and were used here to check network performance.

A single cell information measure considers the maximum amount of information the cell has about any one stimulus / object independently of which transform (e.g. view of the object) is shown. Because the competitive algorithm used in VisNet tends to produce local representations (in which single cells become tuned to one stimulus or object), this information measure can approach $\log_2 N_S$ bits, where N_S is the number of different stimuli. Indeed, it is an advantage of this measure that it has a defined maximal value, which enables how well the network is performing to be quantified. High information measures show that cells fire similarly to the different transforms of a given stimulus (object), and differently to the other stimuli. The single cell stimulus-specific information, $I(s, R)$, is the amount of information the set of responses, R, has about a specific stimulus, s (see [41, 9]). $I(s, R)$ is given by

$$I(s, R) = \sum_{r \in R} P(r|s) \log_2 \frac{P(r|s)}{P(r)} \tag{11}$$

where r is an individual response from the set of responses R of the neuron. For each cell the performance measure used was the maximum amount of information a cell conveyed about any one stimulus. This (rather than the mutual information, $I(S, R)$ where S is the whole set of stimuli s), is appropriate for a competitive network in which the cells tend to become tuned to one stimulus. ($I(s, R)$ has also been called the stimulus-specific surprise [42, 31]. Its average across stimuli is the mutual information $I(S, R)$.)

A multiple cell information measure is also used with VisNet, as follows. If all the output cells of VisNet learned to respond to the same stimulus, then the information about the set of stimuli S would be very poor, and would not reach its maximal value of $\log_2$ of the number of stimuli (in bits). The second measure that is used here is the information provided by a set of cells about the stimulus set, using the procedures described by [43, 9]. The multiple cell information is the mutual information between the whole set of stimuli S and of responses R calculated using a decoding procedure in which the stimulus s' that gave rise to the particular firing rate response vector on each trial is estimated. (The decoding step is needed because the high dimensionality of the response space would lead to an inaccurate estimate of the information if the responses were used directly, as described by [43, 14].) A probability table is then constructed of the real stimuli

s and the decoded stimuli s'. From this probability table, the mutual information between the set of actual stimuli S and the decoded estimates S' is calculated as

$$I(S, S') = \sum_{s,s'} P(s, s') \log_2 \frac{P(s, s')}{P(s)P(s')} \tag{12}$$

This was calculated for the subset of cells which had as single cells the most information about which stimulus was shown. In particular, in [9] and subsequent papers, the multiple cell information was calculated from the first five cells for each object that had maximal single cell information about that object, that is from a population of 50 cells if there were ten stimuli (each of which might have been shown in for example 9 views).

Full details and code for these information theoretic measure are provided elsewhere [3, 5].

In the research described here, an Object Selectivity measure was also used, to assess how well a trained network responded differently to all objects, and to all transforms of each object. This object selectivity measure was derived from the correlation matrices between stimuli, illustrated for example in Fig. 2 of the main text [1]. The object selectivity measure was the correlation between the different transforms of an object, divided by (the optimal correlation between the transforms of an object + the responses to any other objects). The maximum value for perfect view invariance for each object and no response to any other object is 1.0, and the minimal value is 0.

References

[1] C. Zhang, E. T. Rolls, and J. Feng. Invariant visual object and face learning in the ventral cortical visual pathway: a biologically plausible model. *PLoS Computational Biology*, page doi: 10.1371/journal.pcbi.1013959, 2026.

[2] G. Wallis and E. T. Rolls. Invariant face and object recognition in the visual system. *Prog Neurobiol*, 51:167–94, 1997.

[3] E. T. Rolls. Invariant visual object and face recognition: neural and computational bases, and a model, visnet. *Front Comput Neurosci*, 6:35, 2012.

[4] E. T. Rolls. Learning invariant object and spatial view representations in the brain using slow unsupervised learning. *Frontiers in Computational Neuroscience*, 15:686239, 2021.

[5] E. T. Rolls. *Brain Computations and Connectivity.* Oxford University Press, Open Access, Oxford, 2023.

[6] E. T. Rolls. Neurophysiological mechanisms underlying face processing within and beyond the temporal cortical visual areas. *Philosophical Transactions of the Royal Society*, 335:11–21, 1992.

[7] P. Földiák. Learning invariance from transformation sequences. *Neural Computation*, 3: 193–199, 1991.

[8] G. Wallis, E. T. Rolls, and P. Földiák. Learning invariant responses to the natural transformations of objects. *International Joint Conference on Neural Networks*, 2:1087–1090, 1993.

[9] E. T. Rolls and T. Milward. A model of invariant object recognition in the visual system: learning rules, activation functions, lateral inhibition, and information-based performance measures. *Neural Comput*, 12:2547–72, 2000.

[10] E. T. Rolls and S. M. Stringer. Invariant object recognition in the visual system with error correction and temporal difference learning. *Network*, 12:111–29, 2001.

[11] E. T. Rolls. Functions of the primate temporal lobe cortical visual areas in invariant visual object and face recognition. *Neuron*, 27:205–218, 2000.

[12] E. T. Rolls and G. Deco. *Computational Neuroscience of Vision.* Oxford University Press, Oxford, 2002.

[13] E. T. Rolls and M. J. Tovee. Processing speed in the cerebral cortex and the neurophysiology of visual masking. *Proceedings of the Royal Society, B*, 257:9–15, 1994.

[14] E. T. Rolls and A. Treves. *Neural Networks and Brain Function.* Oxford University Press, Oxford, 1998.

[15] Y. Miyashita. Neuronal correlate of visual associative long-term memory in the primate temporal cortex. *Nature*, 335:817–820, 1988.

[16] Y. Miyashita. Perirhinal circuits for memory processing. *Nat Rev Neurosci*, 20:577–592, 2019.

[17] P. Rhodes. The open time of the NMDA channel facilitates the self-organisation of invariant object responses in cortex. *Society for Neuroscience Abstracts*, 18:740, 1992.

[18] P. Foldiak. Models of sensory coding. Technical Report CUED/F–INFENG/TR 91, University of Cambridge, Department of Engineering, Cambridge, 1992.

[19] N. Spruston, P. Jonas, and B. Sakmann. Dendritic glutamate receptor channel in rat hippocampal CA3 and CA1 pyramidal neurons. *Journal of Physiology*, 482:325–352, 1995.

[20] S. Hestrin, P. Sah, and R. Nicoll. Mechanisms generating the time course of dual component excitatory synaptic currents recorded in hippocampal slices. *Neuron*, 5:247–253, 1990.

[21] A. M. Houben and M. S. Keil. A calcium-influx-dependent plasticity model exhibiting multiple stdp curves. *J Comput Neurosci*, 48:65–84, 2020.

[22] R. S. Sutton and A. G. Barto. Towards a modern theory of adaptive networks: expectation and prediction. *Psychological Review*, 88:135–170, 1981.

[23] C. von der Malsburg. Self-organization of orientation-sensitive columns in the striate cortex. *Kybernetik*, 14:85–100, 1973.

[24] D. E. Rumelhart and D. Zipser. Feature discovery by competitive learning. *Cognitive Science*, 9:75–112, 1985.

[25] J. Hertz, A. Krogh, and R. G. Palmer. *Introduction to the Theory of Neural Computation.* Addison-Wesley, Redwood City, CA, 1991.

[26] D. J. Willshaw and C. von der Malsburg. How patterned neural connections can be set up by self-organization. *Proceedings of the Royal Society of London B*, 194:431–445, 1976.

[27] S. Grossberg. Adaptive pattern classification and universal recoding: Ii. feedback, expectation, olfaction, illusions. *Biol Cybern*, 23:187–202, 1976.

[28] E. Oja. A simplified neuron model as a principal component analyser. *Journal of Mathematical Biology*, 15:267–273, 1982.

[29] D. Van Essen, C. H. Anderson, and D. J. Felleman. Information processing in the primate visual system: an integrated systems perspective. *Science*, 255:419–423, 1992.

[30] L. Franco, E. T. Rolls, N. C. Aggelopoulos, and J. M. Jerez. Neuronal selectivity, population sparseness, and ergodicity in the inferior temporal visual cortex. *Biological Cybernetics*, 96: 547–560, 2007.

[31] E. T. Rolls and A. Treves. The neuronal encoding of information in the brain. *Progress in Neurobiology*, 95:448–490, 2011.

[32] J. E. Hummel and I. Biederman. Dynamic binding in a neural network for shape recognition. *Psychological Review*, 99:480–517, 1992.

[33] J. Buhmann, J. Lange, C. von der Malsburg, J. C. Vorbrüggen, and R. P. Würtz. Object recognition in the dynamic link architecture: Parallel implementation of a transputer network. In B. Kosko, editor, *Neural Networks for Signal Processing*, pages 121–159. Prentice Hall, Englewood Cliffs, NJ, 1991.

[34] K. Fukushima. Neocognitron: a self-organizing neural network model for a mechanism of pattern recognition unaffected by shift in position. *Biological Cybernetics*, 36:193–202, 1980.

[35] G. Deco and E. T. Rolls. A neurodynamical cortical model of visual attention and invariant object recognition. *Vision Research*, 44:621–644, 2004.

[36] J. Daugman. Complete discrete 2D-Gabor transforms by neural networks for image analysis and compression. *IEEE Transactions on Acoustic, Speech, and Signal Processing*, 36:1169–1179, 1988.

[37] D. Pollen and S. Ronner. Phase relationship between adjacent simple cells in the visual cortex. *Science*, 212:1409–1411, 1981.

[38] R. L. De Valois and K. K De Valois. *Spatial Vision.* Oxford University Press, New York, 1988.

[39] T. S. Lee. Image representation using 2D Gabor wavelets. *IEEE Transactions on Pattern Analysis and Machine Intelligence*, 18,10:959–971, 1996.

[40] D. L. Ringach. Spatial structure and symmetry of simple-cell receptive fields in macaque primary visual cortex. *Journal of Neurophysiology*, 88:455–463, 2002.

[41] E. T. Rolls, A. Treves, M. Tovee, and S. Panzeri. Information in the neuronal representation of individual stimuli in the primate temporal visual cortex. *Journal of Computational Neuroscience*, 4:309–333, 1997.

[42] M. R. DeWeese and M. Meister. How to measure the information gained from one symbol. *Network*, 10:325–340, 1999.

[43] E. T. Rolls, A. Treves, and M. J. Tovee. The representational capacity of the distributed encoding of information provided by populations of neurons in the primate temporal visual cortex. *Experimental Brain Research*, 114:149–162, 1997.
