# Supplementary material for: Invariant visual object and face learning in the ventral cortical visual pathway: A biologically plausible model
Source: PLoS Comput Biol. 2026 Feb 11;22(2):e1013959. doi: 10.1371/journal.pcbi.1013959 (PMC12916060; doi:10.1371/journal.pcbi.1013959)
Supplement: S1 Code — (ZIP) [file pcbi.1013959.s002.zip › VisNetMat3code/VisNetImageFiltering/gaborL3/ffw/readme.pdf]

FFW - Fastest Filtering in the West

The FFW package is an FFT-based algorithm for a fast 2D convolution using the overlap-add method. The overlap-add method is based on the fundamental technique in DSP: decompose the signal into simple components, process each of the components in some useful way, and recombine the processed components into the final signal. This is possible since the convolutional operator is linear. The FFW package works similarly to fftfilt function (Matlab Image Processing Toolbox) but in a deeper way: all possible lengths for vectors are considered and **not only lengths which are powers of two**. This is highly necessary since the FFTW package (for more details visit http://www.fftw.org/) includes codelets optimized also for other fixed sizes. Codelets are produced automatically by the FFTW codelet generator: you can add your own codelets and re-calculate the execution times for each FFT.

The execution times for:

- FFT of real 1D vectors
- FFT of complex 1D vectors
- IFFT of complex 1D vectors

have been calculated with the script provatempo2.m from length N = 3 up to length N = 2048. A finer determination of such times can be done using PAPI for Matlab (available at http://www.mathworks.com/matlabcentral/fileexchange/loadFile.do?objectId=5445&objectType=File or http://icl.cs.utk.edu/papi/).

A 2D FFT (see Matlab command fft2) is decomposed into several 1D FFTs: the FFT operator for an N-dimensional array can in fact be splitted into several 1-dimensional FFTs of monodimensional arrays. The FFW algorithm automatically selects which is the best choice (first dimension, second dimension and best lengths for overlap-add method) and calculates the 2D convolution.

The FFW package can be easily used to improve speed performances of:

- 2D convolution (Matlab function conv2)
- 2D filtering (Matlab function filter2)
- 2D cross-correlation (Matlab function xcorr2)
- normalized cross-correlation (Matlab function normxcorr2)

See test_main.m for many examples of the FFW package.

Please contribute if you find this software useful.
Report bugs to luigi.rosa@tiscali.it

Luigi Rosa
Via Centrale 35
67042 Civita Di Bagno
L'Aquila – ITALY
mobile +39 320 7214179
email luigi.rosa@tiscali.it
web site http://www.advancedsourcecode.com
