# Supplementary material for: Invariant visual object and face learning in the ventral cortical visual pathway: A biologically plausible model
Source: PLoS Comput Biol. 2026 Feb 11;22(2):e1013959. doi: 10.1371/journal.pcbi.1013959 (PMC12916060; doi:10.1371/journal.pcbi.1013959)
Supplement: S1 Code — (ZIP) [file pcbi.1013959.s002.zip › VisNetMat3code/VisNetMat3Doc25.pdf]

File: VisNetMat3Doc25.docx: Updated 9 December 2025

VisNetMat3 is a Matlab-only tutorial implementation of VisNet3 made available with
Zhang,C Rolls,ET and Feng,J (2026) Invariant visual object and face learning in the ventral cortical visual pathway: a biologically plausible model. PLoS Computational Biology and with
Rolls, E. T. (2026) Brain Computations and principles; and AI. Oxford University Press: Oxford.

The code is written by Edmund Rolls, and is made available for academic use with download at https://www.oxcns.org.

The previous version is VisNet1 made available as VisNetMat1Cv1b.zip with (Rolls 2021) and with Rolls (2023) Brain Computations and Connectivity. Oxford University Press.

For the background literature see (Rolls 2012, 2021, 2023, 2026; Zhang *et al.* 2026).

© copyright E T Rolls, Oxford Centre for Computational Neuroscience, https://www.oxcns.org
No warranty express or implied is provided: this is academic research software.
If this software is used or adapted for new published research, it is requested that the original publications, which provide details, are cited.

The main differences between VisNet1 and VisNet3 are that VisNet3 adds new learning rules selected with NORM and optional clipping of the maximal values of synaptic weights using MAX_WEIGHTS as described in Zhang, Rolls and Feng (2026). An associative update of the synaptic weights is the default in train.m.

1. VisNetMat3
This is a Matlab-only version of VisNet mainly for tutorials / teaching / demonstration.
The current version is in /VisNetMat3
For VisNetMat3, the files are VisNetDriver3.m, CreateNet.m, SetIMLIST.m, train.m, test.m, info.m, DisplaySynWeights.m, ObjectSelectivity.m, gui.m and gui.fig.
The information analysis routines infors_visnet.m and inform_visnet.m and the function phims.m are provided in /VisNetMat2.
The images are in \imagedata\

In **VisNetMat3**, run VisNetDriver3.m to test the system. This program expects to find the images in base_dir, which needs to be specified in setIMLIST.m along the following lines:
base_dir = '..\imagedata\128image_9\'; % this has just the first 9 objects
or wheherever else you keep imagedata, e.g.
base_dir = 'c:\imagedata\128image_9\'; % this has just the first 9 objects

For testing with larger numbers of objects, code is made available as described below in ‘2. Image preparation using Gabor filtering’.
In either case, you need to modify the path in the SetIMLIST.m file to enable the \imagedata directory to be found on your computer.

1.1 To set up VisNetMat3 for the first time the following is suggested:
Unzip VisNetMat3code.zip. It contains 4 folders, and VisNetMat3Doc25.pdf

Change folder to get into VisNetMat3code\VisNetMat3
Start Matlab and in VisNetMat3code\VisNetMat3 run with Matlab VisNetDriver3.m
VisNetDriver3.m should run and take a few minutes to train, and then show you the Object Selectivty measure and a correlation matrix for the layer 4 firing across all objects and views. You should see diagonal squares as shown in Fig. 2 of (Zhang *et al.* 2026).

1.1.2 Further information.

Folder imagedata should contain subfolders /testbars256 and /128image_9, which are filtered image folders for 3 bars with 3 views; and 9 objects ALOI objects (Geusebroek et al. 2005) with 9 views respectively. The filtering has been performed using the code in /VisNetImageFiltering to produce Gabor-filtered representations of the images similar to what is represented in the primary visual cortex, V1.

1.1.3 In Matlab, edit the base_dir in SetIMLIST.m as described above.

Note: Windows 11 uses '\' in paths, and linux uses '/' in paths. The code is set up for Windows 11. If you are using a different type of operating system, you may need to alter '\' to '/' throughout the code.
This applies to Macs, which should be set up to use a linux-style path.
Check this wherever a directory path is shown in all .m files.

1.1.4 Make sure that in VisNetDriver3.m, near line 73,
CreateNetFlag = 1;
This will make sure that a new net with random weights and connections such as netUntrained32.mat is built in /VNanalysis/
On future runs, to save time building the net again, you can set
CreateNetFlag = 0;
But for quantitative studies it is better to create a new net each time, with the random number generator set “rng('shuffle');” near line 78 of VisNetDriver3.m

1.1.5 /imagedata may contain both /testbars256 and /128image_copy
In SetIMLIST.m, set ImageSet=1 for /testbars256, and ImageSet=2 for /128image_9.

1.1.6 When you run VisNetDriver3.m, it should run and produce 100% correct and ObjectSelectivity close to 1 for ImageSet=1, and for ImageSet=2. If it does not, check the above.
Next, try the gui (graphical user interface) by typing 'gui' in the Matlab command window to explore the operation of VisNet.

The ObjectSelectivity measure takes a maximum value of 1.0 when all the views of any object are highly correlated with each other, and are orthogonal to the views of all other objects. The function also displays a correlation matrix, which shows the correlations first between all views of object 1, then all views of object 2, etc.
A view here corresponds to a transform.
To measure the capacity, a plot could be made of the ObjectSelectivity or the Percent Correct as a function of the number of objects in the training set.
A criterion for the capacity could be selected, such as the number of objects in the training set at which the ObjectSelectivity decreases below e.g. 0.8, or the Percent Correct drops below 80%.
Note that the Object Selectivity measure takes the rate of all neurons in a layer into account. In contrast, the Information and Percentage correct take into account the 5 most selective and invariant cells for each object.
The multiple cell information analysis program inform_visnet.m is called by VisNetDriver3.m via info(4), and shows the Percent Correct and Smoothed information I_p as another measure of performance. The program inform_visnet.m by default shows the information based on the 5 best cells for all the objects defined by N_GROUPS (which sets the number of objects), but can be edited to show the information for all 1 to 5 * N_GROUPS cells.

1.2 The Graphical User Interface (GUI) to look at the outputs produced by VisNet3.
After a run has finished, the GUI can be started by
gui
and the files are gui.m and gui.fig built using Matlab guide (guide(‘gui.fig’)).
In the gui, do not alter 'Loc' from its value of 1, for translation to different locations using code is not implemented in VisNet3 and needs to be learned.

Clicking on neuron in the layer firing rate window (top left) will set X, Y and the cell number (in the 32x32 matrix of firing rates for a layer depending if NET_SIZE=32) in their boxes, and will update the firing display.
Setting a cell number in its box, then clicking Update, will update X and Y in their boxes, and will update the neuron firing rate display. Update should be used when changing a layer, view etc.
All gui coordinates, group, loc and layer numbers start at 1, which corresponds to the numbering used in Matlab.

In the Rates vs Transforms window for a cell, the order is:

Transform1 Transform2 etc, where each transform is a different view etc

The Dot Product measure shown in the terminal output is a dot product matrix showing the correlations between the stimuli in the firing rate vector to each stimulus. In the diagonal, a value of 1.0 reflects perfect rates of 1.0 to every transform of that stimulus. The rates are also saved to NeuronRates.mat.
For the cell locations within a layer shown as X and Y in the gui, 1,1 is the top left and 128,128 (or whatever NET_SIZE is set to) is the bottom right.
If the gui is resized, the X,Y coordinates may not be returned correctly from the layer firing rate display (top left).

[The following is not implemented in VisNetMat3: If the Layer is 1, the Point (bottom left), Gauss (second from left), and FilterSum (third from left) images are updated. Clear will clear what is being accumulated by successive updates in these 3 windows.]

[If programming the gui, it is useful to close the gui (by clicking on the icon in the top left of the gui window), so that the altered code in gui.m starts afresh.
Guide writes to gui.fig to place the boxes etc in the gui window.

1.3 Parameters that can be changed to investigate how they affect performance:

NET_SIZE: This can be set to 16, 32, 64 128 or 256.
This enables investigation of how the size of the network affects the performance.
Suitable performance measure are the ObjectSelectivity, the Percent Correct, and the Multiple cell information, which should be close to log_2(NumberofObjects) if the system is categorizing the Objects separately and in a transform-invariant way.
The first time that a new NET_SIZE is investigated, CreateNetFlag should be 1 to create a new UntrainedNetwork in ..\VNanalysis. After that, to save the net creation time, CreateNetFlag
can be set to 0, and the UnTrainedNet will be read in.
However, for NET_SIZE=16, or 32 CreateNetFlag should be always be 1, as small nets are fast to create.
However, for systematic unbiased analyses, it is good to create a new network each time, and make sure a new random number is being selected in VisNetDriver3.m (set “rng('shuffle');” near line 76 of VisNetDriver3.m). The average of several runs can then be used.

TrainEpochs = [20 20 20 20]; should normally be used to allow convergence during the learning, but can be reduced to say [7 7 7 7)] for rapid exploration of parameters. High values of TrainEpochs and low values of LRN_RATE may produce better performance.

NSYN = [340 200 200 200]; % synapses per neuron; sets the number of synapses per neuron for Layers 1-4. The 340 should be left unchanged unless you alter NSYNL1 too. For layers 2-4, higher capacities can normally be obtained by increasing this number to 500 or even 1000. With a small net, if the number of synapses is set too high, the net cannot be created, as a high number of synapses may not fit into a small radius of connectivity to the preceding layer.

SPARSENESS = [0.01 0.01 0.01 0.01]; % the sparseness for each layer. Works for NET_SIZE 32 to 256. This sets the sparseness of the firing. Too low a value will make VisNet a look-up table; too large a value will prevent VisNet from discriminating between the stimuli, because the firing rates to the different stimuli will be somewhat similar.

N_GROUPS = 9; % number of objects. Alter this to measure how the performance depends on the number of objects; and test how this varies with different NET_SIZE values.

N_VIEWS = 9; % number of transforms of each object for learning : this should ideally be set to the number of transforms; BUT if there are fewer transforms available, each of the transforms can be repeated several times, to produce a list of 9 (or more), as a list of this length is required for trace rule learning. During training, VisNet selects a random set of 5 of the views of an object and presents them to allow the trace of previous firing to build up, and then the N_VIEWS are presented with synaptic modification to allow learning.

N_VIEWS_TEST = 9; % number of transforms for testing: 9 for objects, 3 for bars : this is normally the same as **N_VIEWS,** but if the actual number of views is fewer as for ImageSet =1, then N_VIEWS_TEST should be reduced to the number of different views / transforms.

ImageSet can be 1 (bars) or 2 (Amsterdam Library of Images, ALOI (Geusebroek *et al.* 2005), objects); but you can create new image sets.

SIGMOID = 1; % set this to 1 for sigmoid activation function, 0 for linear threshold

BETA = [10 10 10 10] * 1; % beta, the slope of the sigmoid activation function for the neurons in each layer.

LATERALINHIB = 1; % 1 for convolution of the firing rates in a layer with a lateral inhibition filter. 0 for none. The lateral inhibition filter has a width of 0.2 and a sd of 4 with a Sum of zero, so that a neuron inhibits its neighbours to help a diversity of representations to be learned. The filter is in dog1_0.2_4.mat, and was created with the program dogeg.m

ETA = [0.0 0.8 0.8 0.8]; % the trace value for each layer : a higher value increases the proportion of the previous firing in the trace used for the learning, relative to the firing being contributed by current inputs. Increasing this value will mean that more training epochs are needed.
No trace is used for layer 1, which utilizes associative learning only.
In train.m, the code for Layers 2-4 is to use the trace from the preceding trials but not the current trial, so even if ETA for these higher layers is set to 0, there will be some trace-rule learning. There are options to use a trace value that includes the current image being shown, and if this is edited in in train.m, values of zero for ETA for the higher layers should produce no transform-invariant learning.

LRNRATE = [0.1 0.1 0.1 0.1] *0.5; % for the synaptic modification rule (default 0.1) : The amount by which the synaptic weights can change in a single epoch of training. A lower value will mean that more training epochs are needed. A higher value will result in the risk that recently trained stimuli over-write what has been learned about previous stimuli in the list.

If the standard associative learning rule (the default) at about line 289 in train.m is in use, the learning rate can be = [0.1 0.1 0.1 0.1] * 0.5 with few objects, but should normally be lower, and should be decreased if many objects are being trained, so that later object synapses do not overwrite those for other objects already trained in that epoch.

If the error correction rule at about lines 290-291 in train.m is in use, the learning rate that work best may be different.

Some evidence about convergence can be obtained by setting Display=2, and then the change of synaptic weights on each trial will be shown.

(The following two are new in VisNet3, and are described in Zhang, Rolls and Feng (2026).)

NORM This sets the learning rule and synaptic weight normalization being used.
NORM=1; Hebbian associative increase in synaptic weights followed by normalisation of the synaptic weight vector on each neuron. This was the implementation in earlier versions of VisNet.
NORM=2; Oja rule (Oja 1982; Hertz et al. 1991) which implements some weight decay and also may normalise the synaptic weight vector of each neuron.
NORM=3; Hebbian associative increases in synaptic weights and heterosynaptic Long-Term Depression that depends on the existing strength of the synapse (Zhang, Rolls and Feng (2026)). This is the default for VisNet2.
NORM=4; This is Hebbian learning without synaptic wight normalisation to enable investigation of use of MAX_WEIGHTS with only Hebbian associative learning.

MAX_WEIGHTS. This clips the maximum value of the synaptic weights at the value specified for each of the 4 layers, in train.m.
MAX_WEIGHTS[0 0 0 0]; If 0 like this, no weight clipping occurs, the default. In this case the maximum value that could be reached is 1 if NORM=3.
MAX_WEIGHTS[0.1 0.1 0.1 0.1]; In this case the maximum weight for a synapse is clipped at 0.1, and this may improve performance by leading to more distributed synaptic weight vectors in loaded networks.

1.4. Notes

It is instructive after a run to type 'info(3)', 'info(2)', and 'info(1)' to show that the object selectivity becomes lower towards earlier layers, as expected for VisNet3 given that it is a hierarchical network with convergence from layer to layer. The amount of convergence is set for each layer by:
receptiveFieldWidth = [15 7 7 7]; % the width of the Gaussian region from which a neuron receives connections. This is automatically scaled for different NET_SIZEs. These receptive field widths can be changed, and can be explored in the gui, with ImageSet=1 useful for this.
Setting receptiveFieldWidth(1) = 3; may improve performance by maintaining relatively small high-spatial resolution receptive fields in layer 1. If this number is too small, the net will not build, because CreateNet cannot find the number of different synapses specified with a small receptive field width. (In this case the program will hang, and NSYN must be decreased.)

VisNetMat1C (the earlier version) has been tested with up to NET_SIZE=256 x 256 = 64,768 neurons per layer (259,072 neurons across the 4 layers) and 5000 synapses per neuron (NSYN = [272 5000 5000 5000]). (Layers 2-4 between them have 971,520,000 synapses (approximately 10^9.)

N_FILTER_FREQS = 4; % or 8. The default is 4, as this is biologically realistic, and prevents too much apparent translation invariance in Layer 1.
The gui uses /analysis/Sweep.mat, so make sure that Sweep.mat is from the run being analyzed.

If the program fails at the information analysis stage, check that infors_visnet.m sets up appropriate numbers for
max_c = 65536; % /* max no of cells, was 1024, then 16384, then 65536 for 256 * 256 VisNet */
max_s = 50; % /* max no of stimuli*/

Images are filtered with FilterConv4.m in gaborL, which adds to FilterConv3.m the production of a single file with the extension .filt that contains all the filtered component images.

[The program VisNetMat3/CreateImages.m shows how to create simple images such as horizontal and vertical bars. The images then need to be filtered. The filtered images used are 512x512 for each component, for all NET_SIZE (16, 32, 64, 128 and 256).]

For 32x32 VisNetMat2, and 512x512 filtered files, run time is 164 s on a Windows 11 Desktop for 7 epochs, 9 objects, 9 views and N_SYN[340 200 200 200], including all the testing and analysis, and display of results during training with Display=2.

This includes creating a Net, which is built in VisNetDriver3.m if CreateNetFlag=1.
For 128x128 VisNet, and 512x512 filtered files, run time is 792 s on a Windows 11 Desktop for 7 epochs, 9 objects, 9 views and N_SYN[340 200 200 200]

The performance of 32x32 is good using no lateral inhibition, and a threshold linear activation function to set the sparseness at 0.05. (Projects could examine the potential advantages of lateral inhibition, and a sigmoid activation function, the Matlab code for which is part of VisNetMat3.)

If training is with fewer than 9 views, then it is suggested that the images need to be replicated in IMLIST which is used for train, with a separate IMLIST_TEST for testing.

2. Image preparation using Gabor filtering:

The test images are created with CreateImages.m and FilterConv4.m.
ALOI images can be cropped, scaled, centred, and contrast adjusted then filtered with ALOIprocessing3_v4.m. That shows effectively how to take an image in any format, and prepare it for use with VisNet. Blenderprocessing.m can be used if the images are created with Blender.

The software is in VisNetImageFiltering/gaborL3/ which can conveniently be copied into the imagedata/ directory on the computer, which can also contain a directory such as /BlenderIm/ for images produced by FilterConv4.m in /gaborL3/ .) . Note that FilterConv4.m now replaces FilterConv3.m.

Example of use: to filter a new image, e.g. face01L3, which is a 256 * 256 image in a flat file of uint8 (i.e. unsigned bytes in the range 0-255):
place the image into /imagedata/ImageDir (which you need to make, and or wherever you wish for the base directory as specified in the call to FilterConv4),
edit VisNetImageFiltering/gaborL3/FilterConv4.m to ensure that it shows the correct number of spatial frequencies to compute. The default from 28 Jan 2013 is freqlist = [1 2 4 8].
In matlab, cd VisNetImageFiltering/gaborL3, FilterConv4('face01L3', 'ImageDir', Triples)

where Triples=0 (symmetric filtered output only),
or Triples=1 to have in each row triples of (symmetric, asymmetric, asymmetric+180deg).
(That is column 0 will have the symmetric filter value for column 0
column 1 will have the asymmetric filter_left value for column 1
column 2 will have the asymmetric filter_right value for column 2)
The current recommendation is Triples=0.
e.g. cd VisNetImageFiltering/gaborL3 (and assume there is directory imagedata/bars256 which contains an image vbars256)
FilterConv4('vbars256', 'c:/imagedata/vbars256/',0)

FilterConv4.m can be edited to
Set Display=0 (no Display), or Display=1

Alter the frequencies computed, which are [1 2 4 8 16 32 64 128], or as better and as supplied with [1 2 4 8], appropriate for VisNetMat3 which expects a 256x256 image to be supplied. (The software places the 256x256 grayscale image into a 512x512 blank background to prevent wrap-around effects.)
Fsize, the filter size, is set to 512 for 512x512 filtered images for VisNetMat3. FilterConv4.m expects with Fsize=512 a 256x256 flat 8 bits per pixel (uint8, range 0-255) image to be supplied, which it pads to 512x512 smoothing it into a 127 gray level background. The image supplied should have a mean close to 127, and the contrast for different images in a training set should be similar to each other.

FilterConv4.m uses an fft-based convolution utilizing convolve2() which is fast for the large filter.

/gaborL3/ contains all the gabor filters, already computed for 8 spatial frequencies from .1 up to .128.

(To remake the gabor filters (with the following developed in Linux):
In /VisNetImageFiltering/gaborL3:

Edit run.h to set NLEVELS to e.g. 4 (or 8)

gcc -o3 gaborfast512.c -o gaborfast512 -lm

(rng is not used so OK)

./gaborfast512 image.dat

(the image.dat should be 256x256 but is not used. The program will take many hours to run to produce the .128 filters)

FilterConv4 places all its filtered images into a subdirectory image.filtered/, which also contains the original 256x256 image, and image128.dat which is used for the VisNet display image and which has the image as 64x64 on a 128x128 background of level=127.

In FilterConv4.m set Display=2 to show the for Posim, Negim etc.

TestFiltered.m will display the filtered images as a check, e.g.

TestFiltered('face01L3')

Edit it first to make sure that the paths are correct, and that the Fsize and freqs are as in the filtered images. It is set up for Fsize=512.

dispRawImage('filename', dim) in dim=256 will display a 256 x 256 uint8 image 65536 bytes long such as that used to show in VisNet a picture of a test image.
e.g. dispRawImage('bmwETR_0.0', 256)
It will also create simple test images such as bars (in the current directory).

ALOIprocessing (using ALOIprocessing3_v4.m)

cd VisNetImageFiltering/gaborL3
make sure that there is a directory /imagedata/ALOI containing the ALOI images to process,
edit datapath in ALOIprocessing3_v4.m
edit filenamebase in FilterConv4.m
then
ALOIprocessing3_v4 (in Matlab)
should process all the ALOI images, taking in a typical case 42 s for every view of one ALOI object.
The resizing, image contrast setting, and filtering is implemented in this code. imadjust is used and is better than histeq for setting the image contrast.
ALOI_processing3_v4.m also writes the cropped and contrast adjusted image as a .png to the output as an easy data check.

Processing of Blender Images

/imagedata/BlenderIm contains the objects, each in directories like /1, /2 etc
cd /imagedata/gaborL3
matlab &
BlenderProcessing.m which calls FilterConv4.m in the same directory.

[The rest of this Blender software is not in this directory
To view a set of Blender images:
show_Blender_png_array (for 6 objects)
show_Blender_png_array2 (for 7 objects, and improved display of a whole image)]

Use of Blender software to make test images:

Install blender version 2.66 from blender.org which contains 'cycles' functionality.

Unpack the blender-2.66-linux…tar.bz2, and make sure that all the files are placed in the /home/erolls/blender directory.
(It may be useful to set blender to produce RGBA images with alpha, the transparency layer of a .png file, set, using the option ‘straightalpha, though render.py may set this.)

./blender -b man.blend -P /home/erolls/blender/render.py

This calls /VisNetM3/scaleTranspng.m to scale each transparent .png and then place it on a 127 grayscale background, and FilterConv4.m to filter the images.

Further Blender files including a useful Readme from Tristan Webb are in VISNET_DOCUMENTATION/VisNetMatlabDoc2014/BlenderWebb/]

References

Geusebroek JM, Burghouts GJ, Smeulders AWM. 2005. The Amsterdam Library of Object Images. International Journal of Computer Vision 61:103-112.

Hertz J, Krogh A, Palmer RG. 1991. Introduction to the Theory of Neural Computation. Redwood City, CA: Addison-Wesley.

Oja E. 1982. A simplified neuron model as a principal component analyser. J Math Biol 15:267-273.

Rolls ET. 2012. Invariant visual object and face recognition: neural and computational bases, and a model, VisNet. Front Comput Neurosci 6:35.

Rolls ET. 2021. Learning invariant object and spatial view representations in the brain using slow unsupervised learning. Front Comput Neurosci 15:686239.

Rolls ET. 2023. Brain Computations and Connectivity. Oxford: Oxford University Press, Open Access.

Rolls ET. 2026. Brain Computations and Principles; and AI. Oxford: Oxford University Press.

Zhang C, Rolls ET, Feng J. 2026. Invariant visual object and face learning in the ventral cortical visual pathway: a biologically plausible model. PLoS Comput Biol:in revision.
